# Associations of intelligence across the life course with optimism and pessimism in older age

**DOI:** 10.1016/j.intell.2017.03.002

**Published:** 2017-05

**Authors:** Adele M. Taylor, Stuart J. Ritchie, Ian J. Deary

**Affiliations:** a Department of Psychology, The University of Edinburgh, 7 George Square, Edinburgh EH8 9JZ, UK; b Centre for Cognitive Ageing and Cognitive Epidemiology, University of Edinburgh, 7 George Square, Edinburgh EH8 9JZ, UK

**Keywords:** Cognitive ability, Dispositional optimism and pessimism

## Abstract

Maintaining good cognitive function is important for successful aging, and it has been suggested recently that having and optimistic outlook may also be valuable. However few have studied the relationship between cognitive ability and dispositional optimism and pessimism in older age. It is unclear whether associations found previously between cognitive ability and pessimism in older age, are evident across the life course, and are consistent at different points in older age. In the present study we examined associations between dispositional optimism and pessimism measured in the eighth and ninth decade of life and childhood and older age cognitive ability, and lifetime change in cognitive ability. Participants were two independent narrow-age samples of older individuals with mean ages about 73 (*n* = 847) and 87 (*n* = 220) years from the Lothian Birth Cohorts of 1936 (LBC1936) and 1921 (LBC1921), respectively. Higher cognitive ability in childhood and older-age, and healthier cognitive change across the lifetime were associated with lower pessimism in older age: age-11 IQ (LBC1936: β = − 0.17, *p* < 0.001; LBC1921: β = − 0.29, *p* = 0.001), older-age IQ (LBC1936: β = − 0.18, *p* < 0.001; LBC1921: β = − 0.27, *p* < 0.001), cognitive change (LBC1936: β = − 0.06, *p* < 0.04; LBC1921: β = − 0.15, *p* = 0.05). Cognitive ability was not significantly associated with optimism in bivariate analyses, and after adjustment for covariates had only small associations with optimism and only in the LBC1936. The results are consistent with differential associations between cognitive functions and optimism and pessimism, and indicate that their associations with cognitive ability are similar in the eighth and ninth decades of life.

## Highlights

• We examined associations between cognitive ability and older-age optimism and pessimism.
• Childhood and older-age cognitive ability were negatively associated with pessimism.
• Lifetime change in cognitive ability was negatively associated with pessimism.
• Cognitive ability has similar associations with pessimism in the 8th and 9th decades of life.

Optimism and pessimism are trait-like facets of personality which describe a person's general expectations for good or bad things to happen (Scheier & Carver, 1985). It has recently been argued that “personal resources such as optimism… are integral to ageing well” (Cosco, Brayne, & Stephan, 2014, p. 35). The suggested importance of an optimistic outlook for successful ageing reflects the current emphasis on psychosocial factors (Cosco, Prina, Perales, Stephan, & Brayne, 2014a), and is perhaps unsurprising given the range of outcomes to which optimism has been linked, including social resources, quality of life, and many physical health measures (Carver and Scheier, 2014, Carver et al., 2010). Preservation of cognitive function has long been a constituent in the conceptualisation of successful aging (Rowe & Kahn, 1987). Researchers and laypersons agree that maintaining cognitive function is key to aging well (Bowling, 2007, Cosco et al., 2014c). Though dispositional optimism and cognitive function are both considered to be important in older age, the relationship between these factors has received relatively limited attention, and it is unclear to what extent they relate to one another. Here, we examine the relations between cognitive ability and optimism and pessimism in older age, using data from two independent, narrow-age samples of individuals in their eighth and ninth decades of life.

Individual differences research on personality provides a plausible mechanism through which cognitive ability might be related to optimism and pessimism in older age. There is evidence that personality traits and psychological outlook are malleable throughout life (Mottus et al., 2012, Smith and Baltes, 1997, Mroczek and Spiro, 2003, Roberts et al., 2005), and that these changes may be due to the effects of significant life-stage experiences, such as career success in mid-life, or death of a spouse in later life (Roberts & Mroczek, 2008). Optimism is closely related to other well-established personality traits, including those known as the ‘Big Five’ personality traits (extraversion, emotional stability, agreeableness, conscientiousness, and intellect), and mood factors (Glaesmer et al., 2012, Marshall et al., 1992, Sharpe et al., 2011), and levels of optimism have also been reported to change across the life course (Chopik, Kim, & Smith, 2015). Furthermore, optimism might also be modified by significant life stage experiences. Chopik et al. (2015) found that increasing optimism over 4 years in later life was related to better self-rated health and fewer chronic illnesses measured over the same period. Similarly to physical health in older age, cognitive decline is prevalent in aging populations and is a cause for great concern (Deary et al., 2009). Differences in cognitive ability could thus be considered highly salient to how older age is experienced, and may be related to individual differences in optimism and pessimism in later life.

We are aware of only two studies which have examined associations between cognitive ability and optimism and pessimism. In a study of 57 high school students aged 15, higher intelligence measured using a figure analogy task was associated with greater optimism (standardized beta coefficient = 0.22; Nurmi & Pulliainen, 1991). In a sample of over 7000 adults at a mean age of 68 years, Palgi (2013) found that higher cognitive ability was associated with lower pessimism (unstandardized beta coefficient = − 0.10), but was not significantly associated with optimism. These studies indicate that differences in cognitive ability are related to how optimistic or pessimistic a person rates themselves to be when measured concurrently, and suggest that this relationship could be present in childhood and in older age. It is unclear, however, if the relationship is present across the life course.

It is possible that cognitive ability measured in childhood is associated with later life optimism and pessimism. Cognitive ability is highly stable across the life course (Gow et al., 2011), and the influence of childhood cognitive ability on important later life physical and mental health outcomes is well established (Deary et al., 2004b, Deary et al., 2010). Furthermore, associations between adulthood optimism and pessimism and predictor variables measured decades earlier in childhood have been demonstrated. Childhood SES (Heinonen et al., 2006), exposure to adversity in childhood (Korkeila et al., 2004), and educational achievement (Ek, Remes, & Sovio, 2004), have all been associated with optimism and pessimism in adults. To our knowledge, no study has examined the association between cognitive ability measured in childhood and later-life optimism and pessimism. This is important, because childhood cognitive ability is associated with these aforementioned childhood correlates of adult optimism and pessimism. Using results from a rarely-available, direct measure of cognitive ability from childhood, and from the same test taken over 60 years later, here we were able to examine this question in two separate older-age samples; that is, we were able to assess whether associations between older-age cognitive ability and optimism and pessimism were independent of childhood cognitive ability. This highly unusual dataset also allowed us to measure lifetime cognitive change and test whether amount of change in cognitive ability across the life course is related to optimism and pessimism in later life.

The aim of the present study was to examine the cross-sectional relationship between cognitive ability and optimism and pessimism in the 8th and 9th decades of life. Participants' completion of cognitive ability tests at age 11 and at age 70 or 87 years allowed us to test the hypotheses that childhood cognitive ability, older age cognitive ability, and lifetime change in cognitive ability would be associated with optimism and pessimism in older age. We also tested these associations after adjustment for potentially confounding variables.

## 1 Methods

Ethical permission for the Lothian Birth Cohort 1936 (LBC1936) study protocol was obtained from the Multi-Centre Research Ethics Committee for Scotland (Wave 1: MREC/01/0/56), the Lothian Research Ethics Committee (Wave 1: LREC/2003/2/29), and the Scotland A Research Ethics Committee (Wave 2: 07/MRE00/58). Ethics permission for the Lothian Birth Cohort 1921 (LBC1921) was obtained from the Lothian Research Ethics Committee (Wave 1: LREC/1998/4/183; Wave 3: LREC1702/98/4/183). The research was carried out in compliance with the Helsinki Declaration. Written, informed consent was given by all participants.

### 1.1 Participants

Participants were from the LBC1936 and LBC1921 studies. Comprehensive recruitment and assessment procedures for both studies have previously been reported (Deary et al., 2004a, Deary et al., 2007). In brief, the LBC studies were designed as follow-ups to the Scottish Mental Surveys of 1947 (SMS1947) and 1932 (SMS1932; Scottish Council for Research in Education (SCRE), 1933, Scottish Council for Research in Education (SCRE), 1949), to study a wide range of determinants of individual differences in non-pathological cognitive ageing (Deary, Gow, Pattie, & Starr, 2012). At about age 11, as part of the Scottish Mental Surveys, most participants had completed a test of general intelligence, a version of the Moray House Test (MHT) No.12. Around six (LBC1936) and seven (LBC1921) decades later, participants sat the same test again when they were recruited to the Lothian Birth Cohort studies.

#### 1.1.1 LBC1936

The LBC1936 consists of 1091 individuals (548 males, 543 females), mostly from Edinburgh and the surrounding Lothian area, who were contacted for follow-up testing between 2004 and 2007 (Wave 1), at an average age of 69.5 years (*SD* = 0.8). Data for the current study mostly comes from Wave 2 of follow-up testing, undertaken between 2007 and 2010 (mean age = 72.5 years, *SD* = 0.7; *n* = 866). Between follow-ups the main reasons for attrition were: death (*n* = 39), permanent withdrawal (*n* = 151), and no longer being eligible or loss of contact (*n* = 35).

#### 1.1.2 LBC1921

The LBC1921 consists of 550 individuals (234 males, 316 females), mostly from Edinburgh and the surrounding Lothian area, who were contacted for follow-up testing between 1999 and 2001 (Wave 1), at an average age of 79.1 years (*SD* = 0.6). Data for the current study mostly comes from Wave 3 of follow-up testing, between 2007 and 2008 (mean age = 86.6 years, *SD* = 0.4; *n* = 235). Between follow-ups the main reasons for attrition were: death (after Wave 1: *n* = 88; after Wave 2: *n* = 55), permanent withdrawal (after Wave 1: *n* = 60; after Wave 2: *n* = 7), and other reasons (after Wave 1: *n* = 81 and 2: *n* = 24; Deary, Pattie, & Starr, 2013).

Analyses in the current paper are based on participants who had complete Life Orientation Test-Revised data (LOT-R; Scheier, Carver, & Bridges, 1994) from the first wave of testing at which the LOT-R was administered to each sample (for the LBC1921 this was the only wave at which it was administered). Those with missing or incomplete LOT-R data were excluded (LBC1936: *n* = 12, LBC1921: *n* = 4). Remaining participants with an Mini Mental State Examination score of < 24 were also excluded; this is a commonly-used cut-off indicating possible dementia (LBC1936: *n* = 7, LBC1921: *n* = 11). The samples remaining for analysis were *n* = 847 for LBC1936, and *n* = 220 for LBC1921.

### 1.2 Measures

Participants from both samples were tested at the same clinical research facility, and the measures used and their administration was the same for each.

#### 1.2.1 Childhood cognitive ability

Cognitive ability in childhood was assessed using a version of the Moray House Test (MHT) No. 12, which most participants completed as part of the SMS1932 or SMS1947 (SCRE, 1933, 1949). The MHT is a group-administered psychometric intelligence test that includes verbal reasoning, arithmetic, spatial and other items. It has a time limit of 45 min, and a maximum score of 76. The MHT was validated at age 11 years against the Terman-Merrill revision of the Binet Scales, with which it correlated about *r* = 0.80 (Scottish Council for Research in Education, SCRE, 1949, p. 123). MHT scores for both the LBC1921 and LBC1936 were corrected for age in days at time of testing by saving the residuals from a regression model predicting the score from the age, then separately converted to an IQ-type scale for the full samples (*M* = 100, *SD* = 15). This will be referred to as age-11 IQ.

#### 1.2.2 Older-age cognitive ability

The same version of the MHT which was completed in childhood was administered to the LBC1936 participants at Wave 1 when they were about 70 years old, and to the LBC1921 participants at Wave 3 when they were aged about 87 years. The same scoring and time limit were used as in the age 11 test. Again, MHT scores were corrected for age and converted to an IQ-type scale for each sample (*M* = 100, *SD* = 15). This will be referred to as older-age IQ.

Because the MHT was not administered concurrently to the LOT-R in the LBC1936, we composed an additional factor of general cognitive ability (*g*) at age 73 to use in a sensitivity analysis. *g* was estimated within a confirmatory factor analysis (CFA) of 14 cognitive ability tests, for the LBC1936 participants only. These were four subtests from the Wechsler Adult Intelligence Scale, third UK Edition (WAIS-III-UK Wechsler, 1998a, Wechsler, 1998b): Matrix Reasoning, Block Design, Digit Symbol Substitution, and Symbol Search; five subtests from the Wechsler Memory Scale-III UK (Wechsler, 1998a, Wechsler, 1998b): Logical Memory, Spatial Span forwards and backwards, Digit Span Backwards and Verbal Paired Associates; and 4-Choice Reaction Time (Deary, Der, & Ford, 2001), Inspection Time (Deary et al., 2004), National Adult Reading Test (Nelson & Willison, 1991), Wechsler Test of Adult Reading (WTAR; Wechsler, 2001), and Verbal Fluency (Lezak, 2004; see Deary et al., 2007 for full details of all tests). We used the same hierarchical structure as in previous papers (Tucker-Drob, Briley, Starr, & Deary, 2014), including one residual covariance path between Spatial Span forwards and Spatial Span backwards, and one residual covariance path between Logical Memory and Verbal Paired Associates, which resulted in good fit to the data (*χ*^2^(71) = 268.09, RMSEA = 0.058, CFI = 0.956, TLI = 0.943). Within the model, we tested the relation of *g* to the LOT-R.

#### 1.2.3 Lifetime cognitive change

For the current study we also calculated cognitive change: a measure of change in cognitive ability between age-11 and 70 (LBC1936) or 87 (LBC1921) years. Cognitive change was calculated separately for each cohort by regressing later-life IQ on age-11 IQ and saving the standardized residuals. This reflects change in IQ relative to others resulting from gains in cognitive ability after childhood or decline during the ageing process to age 70/87.

#### 1.2.4 Optimism and pessimism

The LOT–R was included in a booklet containing eleven (LBC1936 Wave 2, mean age 73) or six (LBC1921 Wave 3, mean age 87) questionnaires, mailed to the participants when booking their follow-up assessments. Participants were instructed to bring the completed booklet to their appointment where it was checked by a trained researcher; errors or omissions were corrected before the end of the visit. The LOT-R has six items; three are positively worded (e.g., “In uncertain times I usually expect the best”), and three are negatively worded (e.g., “If something can go wrong for me, it will”). Four additional filler items were not included in scoring. Participants responded to each item on a 5-point Likert-type scale, ranging from *I disagree a lot* (0) to *I agree a lot* (4). There is growing evidence to suggest that, rather than representing opposite ends of a continuum, optimism and pessimism are individual constructs, differentially associated with external variables (Robinson-Whelen et al., 1997, Roy et al., 2010, Räikkönen and Matthews, 2008), and separable at the genetic level (Bates, 2015). For the current study separate factors of optimism and pessimism were measured by independently summing the positively and negatively worded items, with scores for both ranging from 0 to 12. High scores represented greater optimism and greater pessimism.

#### 1.2.5 Covariates

Age (in days at time of testing) and sex were included as covariates in all analyses. Other potentially confounding variables were selected based on their previous associations with childhood or later life cognitive ability and optimism and pessimism. All covariates were measured in the wave concurrent to administration of the LOT-R (Wave 2 of the LBC1936 and Wave 3 of the LBC1921), except for social class which was recorded at Wave 1 of both studies. Social class was based on participant's reports of their own and their father's principal occupation before retirement. We calculated father's social class using the General Register Office's Census 1951 Classification of Occupations (Office, General. Register, 1956) and participant social class using the Office of Population Censuses Surveys, 1980 (Office of Population Censuses & Surveys, 1980) as these coincided approximately with the midpoint of their careers. Married women also reported their husband's social class which was used if it was higher than their own. For the present study, social class was categorized from professional to unskilled labour under the labels I, II, III, IV, and V; lower social class values represent higher status. At clinic visits, participants reported if they had been diagnosed with hypertension, cardiovascular disease, stroke, diabetes, thyroid disorder, cancer, and dementia, from which we calculated total number of illnesses. The 50-item International Personality Item Pool (IPIP; Goldberg, 2001, Gow et al., 2005) was used to measure the Big-Five personality factors: emotional stability (the inverse of neuroticism, a personality trait characterised by a tendency toward anxiety and low mood), extraversion, agreeableness, conscientiousness, and intellect (similar to openness measured in other Five-Factor model questionnaires). There are 10 items for each of the five personality factors. For ease of reading, ‘I’ was added to the beginning of all items that were in sentence fragment form (e.g., “Am the life of the party”). Participants indicated how well each of the 50 items described them on a 5-point Likert-type scale ranging from very inaccurate (0) to very accurate (4). The IPIP was administered as part of the same booklet of questionnaires as the LOT-R. Current mood state was assessed using the Hospital Anxiety and Depression Scale (HADS; Zigmond & Snaith, 1983). The scale has 14 questions, with seven items addressing anxiety, and seven addressing depression. Higher scores reflect increased symptom severity.

### 1.3 Statistical analysis

Statistical analyses were carried out using IBM SPSS Statistics v.22 and R v.3.1.1. Previous examinations of the factor structure of the LOT-R using confirmatory factor analysis reported that a two-factor structure is a superior fit compared to a unidimensional structure (Glaesmer et al., 2012). In both of the cohorts, we used confirmatory factor analysis (CFA) models to test whether a one-factor model or a two-factor model (where the three optimism and the three pessimism items each had their own factor) was the best fit to the data. Pearson's bivariate correlations were used to examine the correlations between optimism and pessimism, cognitive ability variables, and all covariates. Where one variable was continuous and the other was categorical, these were Spearman's rank correlations. To identify differential associations between predictor variables and dispositional optimism and pessimism we used Williams's test, which tests for significant differences in magnitude of correlations between a predictor variable and competing criterion variables (optimism and pessimism) that are themselves correlated. We also used Fisher's *r*-to-*z* transformations to test for potential differences in magnitude of correlations between LBC1936 and LBC1921 samples. No significant differences between cohorts were found for any associations between cognitive ability variable and optimism and pessimism.

Next we used multiple linear regression to examine the association between childhood IQ, older-age IQ, and cognitive change and optimism and pessimism with adjustments for potentially confounding covariates.

Finally, because cognitive ability in youth is highly predictive of cognitive ability in later life, we conducted a mediation analysis to examine whether any association between childhood IQ and pessimism in older age is accounted for by older-age IQ. We tested whether the path from the predictor (age-11 IQ) to the outcome (pessimism), the predictor to the mediator (older-age IQ), and from the mediator to the outcome were significant. Percentage of mediation was calculated using the equation from Iacobucci, Saldanha, and Deng (2007), and the significance of the mediation was tested using bootstrapped confidence intervals (1000 iterations).

## 2 Results

### 2.1 LOT-R dimensionality and reliability

For both LBC1921 and LBC1936, the CFA models showed that a two-factor solution fit substantially and significantly better (Table 1). For the LBC1921, the correlation between the two factors was *r* = − 0.432, *p* < 0.001, and for the LBC1936, it was *r* = − 0.314, *p* < 0.001. Overall, the CFA models provided clear support for treating the two factors—optimism and pessimism—separately in the analyses below. Cronbach's Alpha for optimism was α = 0.67 and for pessimism α = 0.82 in the LBC1936. In the LBC1921, Cronbach's Alpha for optimism was α = 0.58 and for pessimism was α = 0.72.

**Table 1:** Fit statistics for each confirmatory factor analysis model for the Lothian Birth Cohorts of 1921 (LBC1921) and 1936 (LBC1936).

Cohort	Model	*χ*^2^(df)	RMSEA [95% CI]	CFI	TLI	AIC	*p*_diff_
LBC1921	One-factor	60.30 (9)	0.16 [0.12, 0.20]	0.77	0.62	4073.29	–
	Two-factor	7.11 (8)	0.00 [0.00, 0.07]	1.00	1.01	4022.09	< 0.001
LBC1936	One-factor	219.82 (9)	0.17 [0.15, 0.19]	0.86	0.76	14,197.30	–
	Two-factor	28.66 (8)	0.06 [0.03, 0.08]	0.99	0.97	14,008.14	< 0.001

Note: *p*_diff_ refers to the *p*-value for the *χ*^2^ test of the difference between the one-factor and two-factor models for each cohort. RMSEA = Root Mean Square Error of Approximation; CI = Confidence interval; CFI = Comparative Fit Index; TLI = Tucker-Lewis Index; AIC = Akaike Information Criterion.

### 2.2 Subject characteristics

Descriptive statistics for measures of cognitive ability, optimism and pessimism, and all covariates, separated by cohort, are presented in Table 2. Independent *t*-test and chi-squared tests were used to examine differences between the cohorts for all variables. The mean optimism scores were 8.43 in the LBC1936 and 8.45 in the LBC1921 and these scores were not significantly different. The mean pessimism score was 4.04 in the LBC1936 and 4.78 in the LBC1921 (*p* = 0.001, Cohen's *d* = − 0.20). Depression scores were 1.24 points higher in the LBC1921 also (*p* < 0.001, Cohen's *d* = − 0.43). The participants in the LBC1936 scored 2.30 points higher for extraversion than the participants in the LBC1921 (*p* < 0.001, Cohen's *d* = 0.26). For the current samples, mean change in raw MHT scores (from which IQ scores were calculated) between age 11 and age 70 years for the LBC1936 was 15.4 points (SD = 8.56), and between age 11 and 87 for the LBC1921 was 6.09 points (SD = 12.6), and this difference was significant (*p* < 0.001, *d* = 0.86).

**Table 2:** Characteristics of participants from the LBC1936 and LBC1921 studies.

Characteristic	LBC1936 (M ± SD)		LBC1921 (M ± SD)		Cohort comparison (*p*-value)	Cohen's *d*
Optimism	8.43	2.37	8.45	2.42	0.91	− 0.01
Pessimism	4.04	2.99	4.78	3.03	**0.001**	− 0.20
Age (yrs.)	72.5	0.71	86.6	0.41	**< 0.001**	
Males	437		102			
Females	410		118			
Father's social class (%)					**0.001**	
I	7.1		10.2			
II	20.1		26.6			
III	55.6		48.1			
IV	9.3		10.9			
V	7.9		4.3			
Participant's social class (%)					**0.01**	
I	17.9		23.9			
II	37.7		33.4			
III	40.5		39.4			
IV	3.3		2.1			
V	0.6		1.3			
No of illnesses	1.22	1.06	1.08	0.97	0.08	0.20
Emotional stability	25.0	7.72	24.8	7.32	0.73	0.02
Extraversion	21.6	7.25	19.3	7.20	**< 0.001**	0.26
Agreeableness	30.9	5.53	30.8	4.96	0.91	0.01
Conscientiousness	27.8	6.10	27.2	6.05	0.20	0.08
Intellect	23.8	5.93	23.0	5.70	0.07	0.11
Anxiety	4.47	3.12	4.41	3.18	0.80	0.02
Depression	2.60	2.20	3.84	2.50	**< 0.001**	− 0.43

Note. Social classes are categorized as follows: I (professional occupations); II (managerial and technical occupations); III (non-manual and manual skilled occupations); IV (partly skilled occupations); V (unskilled occupations). *p* values for significant cohort differences are shown in boldface.

To examine the influence of missing data due to dropout on participant characteristics we used *t*-tests to compare each of the observed variables for those who completed the study and those who were lost from the study following the first assessment (see Table 3). In both the LBC1936 and LBC1921 samples, there were significant differences between those who completed the study and those who dropped out. Participants who dropped out of the study had lower IQ at age 11(LBC1936: *p* = 0.004, *d* = − 0.30; LBC1921: *p* = 0.04, *d* = − 0.18), belonged to a higher social class category, indicating a less professional job type (LBC1936: *p* = 0.001, *d* = 0.26; LBC1921: *p* = 0.002, *d* = 0.26), scored lower on emotional stability (both *p* = 0.01, *d* = − 0.24), and reported greater current depression symptoms (LBC1936: *p* = 0.01, *d* = 0.20; LBC1921: *p* = 0.01, *d* = 0.22).

**Table 3:** Comparison of participant characteristics between completers and dropouts in the LBC1936 and LBC1921.

Characteristic	LBC1936			LBC1921		
	Completer (866) (M ± SD)	Dropout (225) (M ± SD)	*p*-value	Completer (235) (M ± SD)	Dropout (315) (M ± SD)	*p*-value
Sex			0.05			0.09
Males	448 (51.7)	100 (44.4)		108 (46.8)	126 (39.5)	
Females	418 (48.3)	125 (55.6)		123 (53.2)	193 (60.5)	
Age-11 IQ	101.7 (15.3)	97.4 (13.4)	**0.004**	101.6 (14.2)	98.9 (15.4)	**0.04**
Age (yrs.)	69.5 (0.83)	69.6 (0.83)	0.10	79.1 (0.57)	79.1 (0.59)	0.85
Father's social class	2.92 (0.94)	2.87 (0.94)	0.51	2.68 (0.98)	2.78 (0.89)	0.25
Participant's social class	2.27 (0.83)	2.48 (0.77)	**0.001**	2.11 (0.84)	2.34 (0.90)	**0.002**
No of illnesses	0.95 (1.00)	1.14 (1.04)	**0.01**	0.98 (0.89)	1.03 (0.94)	0.53
Emotional stability	25.0 (7.67)	23.2 (7.40)	**0.01**	25.4 (8.00)	23.5 (8.01)	**0.01**
Extraversion	21.3 (7.05)	21.5 (7.22)	0.82	20.7 (7.47)	20.6 (7.54)	0.84
Agreeableness	31.0 (5.42)	31.8 (5.15)	0.10	31.9 (4.98)	31.6 (5.29)	0.49
Conscientiousness	28.1 (6.08)	29.1 (5.50)	0.05	29.0 (6.09)	28.5 (6.07)	0.35
Intellect	23.9 (5.66)	23.6 (5.53)	0.56	23.8 (5.39)	23.3 (6.32)	0.35
Anxiety	4.83 (3.10)	5.09 (3.46)	0.27	4.85 (3.36)	5.41 (3.23)	0.05

*p*-values for significant cohort differences are shown in boldface.

### 2.3 Bivariate analyses

Bivariate relations between cognitive ability, optimism and pessimism, and all covariates are shown in Table 4. Age-11 IQ and older-age IQ had significant negative associations with pessimism in the LBC1936 and LBC1921 samples with *r*'s in a narrow range from − 0.25 to − 0.31. In both cohorts the childhood and older-age cognitive scores correlated similarly with pessimism tested in older age. In both cohorts, those whose cognitive function had relatively more decline had higher pessimism (*r* = − 0.09 for LBC1936 and − 0.12 for LBC1921; owing to sample size differences, only the former is significant). No association was found between cognitive ability variables and optimism in either sample. Williams's tests confirmed a significant difference in magnitude between optimism and pessimism associations with age-11 IQ and older-age IQ in both samples (*p*'s < 0.001 in the LBC1936, < 0.05 in the LBC1921).

**Table 4:** Correlations between predictor variables and optimism and pessimism in the LBC1936 and LBC1921.

Predictor variables	LBC1936				LBC1921			
	*n*	Optimism	Pessimism	Optimism-pessimism comparison (*p*-value)	*n*	Optimism	Pessimism	Optimism-pessimism comparison *(p*-value)
Age-11 IQ	801	− 0.01	− 0.31***	< 0.001	196	0.02	− 0.28***	0.02
Older-age IQ	842	− 0.03	− 0.30***	< 0.001	190	0.01	− 0.25**	0.04
IQ change	796	− 0.02	− 0.09**	0.24	168	0.01	− 0.12	0.38
Age	847	− 0.03	0.09**	0.30	220	0.03	0.11	0.20
Sex	847	− 0.01	− 0.08*	0.12	220	0.10	− 0.12	0.85
Father's social class	776	0.02	0.08*	0.10	208	0.05	0.10	0.18
Participant' social class	831	− 0.05	0.26***	< 0.001	218	0.02	0.12	0.20
No of illnesses	847	− 0.05	0.14***	0.12	193	− 0.04	− 0.05	0.44
Emotional stability	846	0.49***	− 0.45***	0.38	218	0.47***	− 0.37***	0.28
Extraversion	847	0.31***	− 0.25***	0.27	218	0.30***	− 0.28***	0.84
Agreeableness	847	0.24***	− 0.27***	0.58	216	0.20**	− 0.30***	0.34
Conscientiousness	847	0.26***	− 0.22***	0.47	217	0.24***	− 0.16*	0.45
Intellect	846	0.19***	− 0.23***	0.47	217	0.18**	− 0.23**	0.64
Anxiety	847	− 0.35***	0.31***	0.45	193	− 0.35***	0.20**	0.17

Note. Coding for binary variables was as follows: male = 0, female = 1. Social classes are categorized as follows: I (professional occupations); II (managerial and technical occupations); III (non-manual and manual skilled occupations); IV (partly skilled occupations); V (unskilled occupations). *p < 0.05, **p < 0.01, ***p < 0.001.

Given the differences in the reliability of the optimism and pessimism scales (see above), we disattenuated the correlations using Spearman's (1904, p.90) formula. For these, we assumed a reliability of 0.8 for the IQ measures (though using values of 0.7 or 0.9 made little difference to the results). The correlations with optimism were near-identical (for instance, in the LBC1921 the age 11 IQ-optimism correlation increased in magnitude from 0.02 to 0.03), and the correlations with pessimism increased slightly (for instance, in LBC1936 the age 11 IQ-pessimism correlation increased in magnitude from − 0.31 to − 0.36 after disattenuation). Thus, the differential relation between intelligence and optimism and intelligence and pessimism did not appear to be driven by the differential reliability of the LOT-R subscales.

Small to moderate associations were also found between age, sex, father's SES, participant's SES, number of illnesses, and pessimism in both samples, though only those in the larger LBC1936 sample were significant (absolute significant *r* values from 0.08 to 0.26; Table 4). The correlations between these variables and optimism appeared smaller and were non-significant, but they were not significantly different in magnitude to the correlations with pessimism, except for participant's SES in the LBC1936. All personality and mood variables were significantly and moderately correlated with optimism and pessimism in both samples (absolute *r* values from 0.16 to 0.49). Greater emotional stability, extraversion, agreeableness, conscientiousness, and intellect were related to greater optimism and lower pessimism, whereas greater anxiety and depression were linked to lower optimism and greater pessimism. Of the personality and mood variables, optimism and pessimism were most highly correlated with emotional stability (absolute *r* values from 0.37 to 0.49) and depression (absolute *r* values from 0.22 to 0.43).

Together, the results indicate that dispositional pessimism, but not optimism, had bivariate associations with cognitive ability and its level and trajectory across the life course. Higher IQ, in childhood and older age, was associated with lower pessimism in older age. Greater relative decline in cognitive ability across the life course—from age 11 to age 70 or 87—was also associated with pessimism in later life.

### 2.4 Regression analyses

Table 5 illustrates hierarchical linear regression results by cohort. Models were run separately for optimism and pessimism outcomes, for each of the cognitive ability predictors (age-11 IQ, older-age IQ, and cognitive change), and separately for LBC1936 and LBC1921. To test whether associations between optimism and pessimism and cognitive ability were independent of potential confounding variables, covariates were entered consecutively in a priori-decided blocks. Age and sex were adjusted for in model 1, and were included in all further models. Father's and participant's SES were added in model 2. Model 3 further adjusted for total number of illnesses. The Big Five personality factors and current mood state were added in model 4. To assess if associations with older age-IQ were independent of age-11 IQ, an further model (5) was fitted to the older-age IQ outcome data which adjusted for age, sex, age-11 IQ, father's and participant's SES, total number of illnesses, and personality and mood. An additional series of regression models were run adjusting for the opposing dispositional factor (i.e., adjustment made for optimism when pessimism was the outcome variable and vice versa). All significant associations between cognitive ability and optimism and pessimism were maintained (see Supplementary Tables S1-S8).

**Table 5:** Multiple linear regression results for three cognitive ability measures predicting optimism and pessimism in the LBC1936 and LBC1921.

		Optimism						Pessimism					
Cognitive predictor (*n*)	Model	B	SE B	β	p	Adjusted R^2^	∆ R^2^	B	SE B	β	p	Adjusted R^2^	∆ R^2^
LBC1936													
Age-11 IQ (*n* = 723)													
	1	0.001	0.01	0.01	0.85	− 0.002	0.002	− 0.06	0.01	− 0.28	**< 0.001**	0.10	0.01
	2	< 0.001	0.01	< 0.001	0.99	− 0.01	< 0.001	− 0.05	0.01	− 0.23	**< 0.001**	0.12	0.02
	3	− 0.001	0.01	− 0.01	0.89	− 0.003	0.003	− 0.04	0.01	− 0.22	**< 0.001**	0.13	0.01
	4	− 0.01	0.01	− 0.07	**0.04**	0.33	0.33	− 0.03	0.01	− 0.17	**< 0.001**	0.34	0.22
Older-age IQ (*n* = 756)													
	1	− 0.004	0.01	− 0.02	0.55	< 0.001	0.003	− 0.06	0.01	− 0.30	**< 0.001**	0.11	0.02
	2	− 0.01	0.01	− 0.03	0.47	− 0.003	0.001	− 0.05	0.01	− 0.25	**< 0.001**	0.12	0.02
	3	− 0.01	0.01	− 0.03	0.42	− 0.002	0.002	− 0.05	0.01	− 0.24	**< 0.001**	0.13	0.01
	4	− 0.02	0.01	− 0.12	**< 0.001**	0.33	0.33	− 0.04	0.01	− 0.18	**< 0.001**	0.34	0.21
	5	− 0.02	0.01	− 0.13	**0.004**	0.33	0.34	− 0.03	0.01	− 0.12	**0.007**	0.34	0.21
IQ change (*n* = 718)													
	1	− 0.07	0.10	− 0.03	0.47	− 0.002	0.002	− 0.44	0.12	− 0.13	**< 0.001**	0.04	0.03
	2	− 0.08	0.10	− 0.03	0.44	− 0.004	0.001	− 0.37	0.12	− 0.12	**0.002**	0.08	0.05
	3	− 0.08	0.10	− 0.03	0.44	− 0.003	0.003	− 0.37	0.12	− 0.12	**0.002**	0.10	0.01
	4	− 0.21	0.08	− 0.08	**0.01**	0.33	0.33	− 0.21	0.10	− 0.06	**0.04**	0.32	0.23

LBC1921													
Age-11 IQ (*n* = 157)													
	1	0.01	0.01	0.05	0.52	− 0.01	0.01	− 0.08	0.02	− 0.37	**< 0.001**	0.15	0.02
	2	0.02	0.02	0.10	0.28	− 0.01	0.01	− 0.08	0.02	− 0.37	**< 0.001**	0.15	0.01
	3	0.02	0.02	0.10	0.28	− 0.02	0.001	− 0.08	0.02	− 0.37	**< 0.001**	0.14	0.001
	4	− 0.01	0.01	− 0.07	0.42	0.37	0.40	− 0.06	0.02	− 0.29	**0.001**	0.27	0.16
Older-age IQ (*n* = 172)													
	1	0.01	0.01	0.04	0.64	− 0.004	0.01	− 0.08	0.02	− 0.35	**< 0.001**	0.14	0.03
	2	0.01	0.01	0.06	0.44	− 0.01	0.01	− 0.07	0.02	− 0.33	**< 0.001**	0.13	0.002
	3	0.01	0.02	0.06	0.45	− 0.02	< 0.001	− 0.07	0.02	− 0.33	**< 0.001**	0.13	0.001
	4	− 0.02	0.01	− 0.10	0.16	0.33	0.36	− 0.06	0.02	− 0.27	**< 0.001**	0.24	0.14
	5	− 0.01	0.01	− 0.07	0.41	0.37	0.40	− 0.05	0.02	− 0.21	**0.01**	0.31	0.15
IQ change (*n* = 152)													
	1	0.10	0.22	0.04	0.66	− 0.01	0.01	− 0.61	0.25	− 0.19	**0.02**	0.06	0.04
	2	0.10	0.22	0.04	0.65	− 0.02	0.01	− 0.56	0.25	− 0.17	**0.03**	0.07	0.03
	3	0.10	0.22	0.04	0.64	− 0.02	< 0.001	− 0.56	0.25	− 0.18	**0.03**	0.07	0.001
	4	− 0.12	0.18	− 0.05	0.50	0.37	0.40	− 0.46	0.24	− 0.15	0.05	0.25	0.21

Note. Model 1 = adjusted for age and sex; Model 2 = as model 1 and additionally adjusted for father's and participant's social class; Model 3 = as model 2 and additionally adjusted for total number of illnesses; Model 4 = as model 3 and additionally adjusted for personality and mood. Model 5 = adjusted for age, sex, age-11 IQ, father's and participant's SES, total number of illnesses, personality and mood. Negative adjusted R^2^ values are statistical artefacts which can be considered as zero *p*-values for significant effects are shown in boldface.

#### 2.4.1 Optimism

In both the LBC1936 and LBC1921, no significant associations were found between age-11 IQ, older-age IQ, or cognitive change and optimism in models 1, 2, or 3 which adjusted for age, sex, father's and participant's SES, and total illnesses, and no model significantly predicted optimism. With the inclusion of personality and mood covariates, model 4 explained about 33% of the variance in optimism in LBC1936 and up to 37% in the LBC1921, but associations with each of the cognitive ability variables remained non-significant in the LBC1921 sample. This additional adjustment for personality and mood increased the size of associations between all cognitive predictors and optimism in the LBC1936, and all became significantly negatively associated (age-11 IQ: β = − 0.07, *p* = 0.04; older-age IQ: β = − 0.12, *p* < 0.001; cognitive change: β = − 0.08, *p* = 0.01). An additional model (5) examined the association between older-age IQ and optimism, adjusting for age-11 IQ in addition to all other covariates. In this model, older-age IQ remained a significant predictor of optimism in the LBC1936 only (β = − 0.13, *p* = 0.004), and age-11 IQ was not significantly associated with optimism.

#### 2.4.2 Pessimism

In model 1, which adjusted for age and sex, age-11 IQ, older-age IQ, and cognitive change all had significant negative associations with pessimism in both samples, and together the variables accounted for up to 11% of the variance in pessimism scores in the LBC1936, and 15% in the LBC1921. Standardized beta coefficients for age-11 IQ and older-age IQ ranged from − 0.28 to − 0.37 in the two cohorts, and those for IQ change were − 0.13 for LBC1936 and − 0.19 for LBC1921, with all being significant. Cumulative inclusion of covariates in models 2, 3 and 4 explained up to 34% of the variance in pessimism in the LBC1936, and 31% of the variance in the LBC1921. The associations with cognitive ability were attenuated slightly at each level of adjustment, but even in model 4 which was fully adjusted for age, sex, father's and participant's SES, total illnesses, personality and mood, cognitive ability variables remained significantly associated with pessimism, or were just reduced below the alpha level for significance: age-11 IQ (LBC1936: β = − 0.17, *p* < 0.001; LBC1921: β = − 0.29, *p* = 0.001), older-age IQ (LBC1936: β = − 0.18, *p* < 0.001; LBC1921: β = − 0.27, *p* < 0.001), and cognitive change (LBC1936: β = − 0.06, *p* < 0.04; LBC1921: β = − 0.15, *p* = 0.05). We assessed the independent association between older-age IQ and pessimism in model 5 after adjustment for age-11 IQ in addition to all other covariates. In this model, older-age IQ was only minimally attenuated and remained a significant predictor of pessimism in both samples (LBC1936: β = − 0.12, *p* = 0.007; LBC1921: β = − 0.21, *p* = 0.01), and age-11 IQ also independently contributed to pessimism in both samples (LBC1936: β = − 0.10, *p* = 0.03; LBC1921: β = − 0.21, *p* = 0.03).

In summary, higher age-11 and older-age IQ had significant negative associations with pessimism, such that individuals with higher IQ in childhood and in older age were less pessimistic in older age. In addition, individuals whose IQ increased between childhood and older age, or declined less over this period relative to others, were also less pessimistic in older age. The only change observed as a result of adjusting for covariates in the regression models was a small increase in the size of association between cognitive ability predictors and optimism in the LBC1936, all of which were negative, indicating that higher cognitive ability and healthier cognitive ageing are associated with less optimism. We emphasise that due to the cross-sectional nature of our study we are not in a position to make causal inferences.

Due to a significant difference in pessimism between males and females in the LBC1936, regression analyses separated by sex were conducted for this outcome. The results showed that in models adjusting fully for all covariates, associations with age 70 IQ and IQ change were no longer significant, but were only slightly reduced for females, and IQ change was no longer significant for males but the association was marginally increased (see Supplementary Table S9). An alternative regression model including an interaction effect between sex and IQ change allowed us to retain the full sample size and indicated no significant difference between males and females in the association between IQ change and pessimism (β = 0.02, *p* = 0.64).

### 2.5 Mediation analysis

We tested whether associations between age-11 IQ and pessimism in older age were mediated by older-age IQ in the LBC1936 and LBC1921 samples. Bootstrapped confidence intervals indicated that, in the LBC1936, older-age IQ significantly mediated 42.7% of the relation between age − 11 IQ and pessimism (standardized mediation effect size = − 0.13, bootstrapped 95%CI = [− 0.19, − 0.07]). In the LBC1921, age 87 IQ mediated 31.6% of the relation between age-11 IQ and pessimism at age 87, but this was not statistically significant (standardized mediation effect size = − 0.09, bootstrapped 95%CI = [− 0.19, 0.02]).

### 2.6 Sensitivity analysis

Due to the non-contemporaneous administration of the MHT and LOT-R in the LBC1936, we conducted a sensitivity analysis to test the robustness of our results for older-age IQ in the LBC1936. First we derived a factor of general cognitive ability at age 73 (*g*) from a CFA of 14 neuropsychological tests. All standardized loadings of tests on their respective domain factors (Visuospatial, Speed, Crystallised, and Memory; see Tucker-Drob et al., 2014) were above 0.4; all standardized loadings of the domains on *g* were above 0.7. We then examined the correlations between g and optimism and pessimism. Similarly to associations found between older-age IQ and optimism (*r* = − 0.03, *p* = 0.43) and pessimism (*r* = − 0.30, *p* < 0.001) in the LBC1936 which were significantly different (*p* < 0.001), *g* was found to be non-significantly related to optimism at age 73 (*r* = 0.04, *p* = 0.27), and negatively and highly significantly related to pessimism (*r* = − 0.37, *p* < 0.001). Again, these correlations were significantly different from each other: a model where they were constrained to equality fit significantly more poorly than a model where they were freely-estimated (*χ*^2^(1) = 24.07, *p* < 0.001, ΔAIC = 22.07). Thus, this analysis is consistent with the results from our main analyses using the MHT.

## 3 Discussion

In two narrow-age samples of older adults, we examined lifelong associations between cognitive ability and dispositional optimism and pessimism. Specifically, and unusually, we were able to test whether childhood and older-age cognitive ability, and cognitive change across the life course, were related to optimism and pessimism in the eighth and ninth decade of life. We found that age-11 IQ and older-age IQ, and to a lesser extent relative cognitive increase, were all negatively related to pessimism in later-life, but were only weakly (and mostly non-significantly) related to optimism.

In both samples, negative associations between all cognitive variables and pessimism were maintained in our multivariate analysis, even after adjustment for personality and mood factors which have previously been argued to be indistinguishable from optimism and pessimism (Smith, Pope, Rhodewalt, & Poulton, 1989). Individuals with higher intelligence in childhood and in older age, and those whose IQ increased more, or declined less, relative to others over the life course, were less pessimistic in later life. In addition to this, our mediation analysis confirmed that some, but not all of the association between age 11 IQ and pessimism was potentially explained by IQ in older age (though the overall mediation effect in the LBC1921 sample was not significant). The results highlight the potential importance of higher cognitive ability from early in life and across the life-course for being less pessimistic in later life. Comparison of effect sizes showed that these effects were similar in the eighth and ninth decades of life. The results also showed that cognitive ability related differently to optimism and pessimism. However, due to the correlational nature of our study, we cannot determine causality.

The association found between older-age IQ and pessimism extends and agrees with results of a previous study in a group of older-adults of a similar age to the LBC1936. Palgi (2013) found higher IQ scores were associated with lower pessimism (unstandardized beta coefficient = − 0.10), in a hierarchical regression that adjusted for covariates including demographics and depression symptoms. In the current study, effect sizes from a similar model (model 4), were slightly smaller, (LBC1936: unstandardized beta coefficient = − 0.04; LBC1921: unstandardized beta coefficient = − 0.06), though these are not directly comparable as additional adjustment was made for health and personality traits in the present study. A positive association between higher cognitive ability and greater optimism in childhood has also been reported (Nurmi & Pulliainen, 1991). Though we were unable to directly test this association in the current sample, we did find a negative association between higher childhood IQ and lower pessimism in older age in both samples. The reason for this association with pessimism rather than optimism in the current study is unclear, though it may be a methodological issue; situation-specific measures of optimism, as used in the previous study, have been found to respond differently to dispositional measures of optimism in some cases (Neff & Geers, 2013). Nonetheless, our results are an important extension of this relatively small literature, showing that not only were cognitive ability and pessimism associated when measured contemporaneously, but differences in cognitive ability measured decades earlier in childhood were also associated with differences in pessimism in later life.

The mechanisms underlying the independent associations between childhood-IQ and older-age IQ and pessimism are unclear. The Life Orientation Test scales were originally developed from behavioural self-regulation models, and expectancy-incentive theory (Scheier & Carver, 1985), which is based on the idea that optimists generally expect more favourable outcomes for future goals and are therefore more motivated than pessimists to engage in efforts to achieve them. The association between childhood cognitive ability and dispositional pessimism in older age fits well with this type of model. Higher intelligence in childhood is theorised to confer benefits in terms of occupation, social status and income (Deary et al., 2005), and reduction in the risk of negative health outcomes and distress (Deary et al., 2010, Hart et al., 2004). Accumulation of this type of advantage across the life course may inform expectations for future outcomes and lead to a less pessimistic outlook in later life. However, associations between cognitive ability and pessimism survived adjustment for father's and participant's SES, and number of illnesses in the current study, suggesting that differences in social status and health alone cannot account for this association. The association between older-age IQ and pessimism might be related to social resources and feelings of loneliness in older age. Though these variables were not examined in the current study, other studies of the current samples have found them to be related to cognitive ability and cognitive decline in older age (Gow, Corley, Starr, & Deary, 2013), and to be predictors of perceived satisfaction with life measured in older age (Gow, Pattie, Whiteman, Whalley, & Deary, 2007).

We also found that change in cognitive ability across the life course predicted pessimism in later life. For some, this change will represent a decline in cognitive ability as part of the aging process. Subjective memory complaints predicted lower extraversion and greater neuroticism in 1450 older males (mean age 63 years; Mroczek & Spiro, 2003). Cognitive function and ability to perform tasks of everyday living are reported to change contemporaneously in older age (Tucker-Drob, 2011). Maintaining physical health and cognitive function allows for independence and autonomy, and is cited by older adults as a being critical for a good quality of life in old age (Bowling, 2005). Individuals who suffer less decline in cognitive ability might be less pessimistic than those whose cognitive ability declines over the life course due to the associated preservation of independence and self-sufficiency.

The associations observed here between pessimism and cognitive ability were stronger and more consistent than associations between optimism and cognitive ability. This is consistent with recent research indicating that these traits have distinct genetic influences (Bates, 2015), and previous studies showing their differential associations with depressiveness (Glaesmer et al., 2012), ambulatory blood pressure (Räikkönen & Matthews, 2008), and inflammation (Roy et al., 2010). The importance of cognitive ability in the regulation of positive and negative emotionality may vary. In a meta-analysis by Ackerman and Heggestad (1997), neuroticism (considered to be negative emotionality) was shown to have a modest negative correlation with general intelligence (*r* = − 0.15), whereas extraversion (considered to be positive emotionality) was found to have a lower positive correlation with general intelligence (*r* = 0.08). Though there is evidence supporting the separable associations of optimism and pessimism, it was unexpected that higher cognitive ability would related to both lower optimism and lower pessimism. Speculatively, this may indicate that individuals with higher IQ have a less extreme outlook on the future, being neither greatly optimistic, nor greatly pessimistic (we thank an anonymous reviewer for suggesting this possibility). Alternatively, it may be a spurious finding. No association existed between cognitive ability and optimism in bivariate analysis, and only became apparent after adjustment for personality and mood factors, and in only in the LBC1936 sample. Previously negligible negative associations were increased and became significant (potentially indicating suppression effects), but the magnitude of associations between each of the cognitive variables and optimism remained small, with the largest increase in standardised β of 0.10 for older-age IQ.

Our data make a novel contribution to research on dispositional optimism and pessimism in older age. Access to childhood IQ data in two older-age samples is rare, and to our knowledge, this is the first study to examine the association between the important outcomes of later-life optimism and pessimism and intelligence in childhood and older age, and change in cognitive ability across the life course. Our data were from two large, demographically similar groups, but with a 14-year age difference, allowing us to explore whether or not the predictors of dispositional optimism and pessimism were the same at two points in older age. The current study is also one of few which have examined dispositional optimism and pessimism in relatively healthy older adults. We had access to several putative determinants of optimism and pessimism and we modelled these together to examine confounding effects, and to examine if associations between older-age cognitive ability and optimism and pessimism in later life were independent of childhood cognitive ability.

The limitations of the study include partial reliance on cross-sectional data which makes it impossible to infer causality. Though we interpret the results as showing that differences in cognitive ability measures predicted levels of optimism and pessimism in later life, we cannot rule out the possibility that these associations may alternatively be bi-directional. For example, the results might indicate that individuals' pessimism causes them to score lower on cognitive ability tests. Pessimism is associated with poorer coping strategies and less goal-directed behaviour (Litt et al., 1992, Nes et al., 2009). It is possible that pessimistic children may be less able to engage with educational goals, leading to lower IQ by age 11, and across the life course. The study benefits from access to cognitive ability data from the same test taken in childhood and in older age; however, optimism and pessimism was first measured in these samples in older age, and without multiple measures we cannot explore the lifetime tracking of these facets of personality.

Furthermore, although we included a comprehensive list of covariates in our analyses, comprising socio-demographics, health, personality, and current mood, the possibility for results to be partly or wholly the product of associations with an unmeasured third variable cannot be discounted.

Selection bias means the participants who attended the initial follow-up of the LBC1936 and LBC1921 studies in older age, and those who continue to participate in the studies over time, are likely to be cognitively and physically fitter than the general population. Longitudinal attrition will also introduce bias as a result of missing data. We judge that it is unlikely that the bias caused by selection and attrition is such that participants who completed the studies have different associations between the variables examined here when compared to those who dropped out; however, it is not possible to know the mechanisms by which missing data affect the current results without having assessed those who dropped out. One possibility is that the bias could bring about a restriction of range. Also, childhood socio-demographic variables were based on retrospective data that may be open to recall error. Finally, in the LBC1936 sample, there was a three-year gap between taking the cognitive ability test (MHT) at about age 70 and completing the LOT-R at age 73, by which point cognitive decline might have occurred or progressed; this may have impacted upon the associations between older-age cognitive ability and change in cognitive ability with dispositional optimism and pessimism in this sample. However, due to the stability of cognitive ability across the life course and during old age, and the results of our sensitivity analysis, we consider the effect on the results to be minimal.

## 4 Conclusion

In the present study, using data from the Lothian Birth Cohorts of 1936 and 1921, we found that higher childhood IQ, higher older-age IQ, and healthier lifetime cognitive change were related to lower pessimism in later life, and that these associations are similar when assessed in the early 70s and later 80s. The associations survived the inclusion of many covariates, including socio-demographic, health, mood state, and personality traits. Our findings are consistent with research suggesting that optimism and pessimism may be separable constructs, and indicate that childhood and older-age cognitive ability are associated with lower dispositional pessimism in the eighth and ninth decades of life.

## Appendix A Supplementary data

Supplementary tables S1–S9. Image 1
