# Supplementary material for: Associations of intelligence across the life course with optimism and pessimism in older age
Source: Intelligence. 2017 May;62:79–88. doi: 10.1016/j.intell.2017.03.002 (PMC5466381; doi:10.1016/j.intell.2017.03.002)
Supplement: Supplementary file 1 — Supplementary tables S1–S9. [file mmc1.docx]

Supplementary Table S1. Multiple linear regression results for age-11 IQ predicting optimism and pessimism in the LBC1936.

|  | Optimism | | | | | |  | Pessimism | | | | | |
| --- | --- | --- | --- | --- | --- | --- | --- | --- | --- | --- | --- | --- | --- |
| Model | *B* | *SE B* | *β* | *p* | *Adjusted*  *R^2^* | *∆R^2^* |  | *B* | *SE B* | *β* | *p* | *Adjusted*  *R^2^* | *∆R^2^* |
| Model 1 |  |  |  |  | -.002 | .002 |  |  |  |  |  | .10 | .01 |
| Age 11IQ | .001 | .01 | .01 | .85 |  |  |  | -.06 | .01 | -.28 | **<.001** |  |  |
| Age | .000 | .000 | -.04 | .27 |  |  |  | .001 | .000 | .09 | **.01** |  |  |
| Sex | -.04 | .18 | -.01 | .83 |  |  |  | -.44 | .21 | -.08 | **.04** |  |  |
| Model 2 |  |  |  |  | -.005 | .000 |  |  |  |  |  | .12 | .02 |
| Age 11 IQ | <.001 | .01 | <.001 | .99 |  |  |  | -.05 | .01 | -.23 | **<.001** |  |  |
| Age | .000 | .000 | -.04 | .29 |  |  |  | .001 | .000 | .08 | **.02** |  |  |
| Sex | -.05 | .18 | -.01 | .79 |  |  |  | -.37 | .21 | -.06 | .08 |  |  |
| Father’s social class | .02 | .10 | .01 | .88 |  |  |  | -.02 | .12 | -.01 | .89 |  |  |
| Participant’s social class | -.07 | .12 | -.02 | .58 |  |  |  | .57 | .14 | .16 | **<.001** |  |  |
| Model 3 |  |  |  |  | -.003 | .003 |  |  |  |  |  | .13 | .01 |
| Age 11 IQ | -.001 | .01 | -.01 | .89 |  |  |  | -.04 | .01 | -.22 | **<.001** |  |  |
| Age | .000 | .000 | -.03 | .37 |  |  |  | .001 | .000 | .07 | **.04** |  |  |
| Sex | -.06 | .18 | -.01 | .73 |  |  |  | -.34 | .21 | -.06 | .11 |  |  |
| Father’s social class | .02 | .10 | .01 | .86 |  |  |  | -.02 | .12 | -.01 | .86 |  |  |
| Participant’s social class | -.07 | .12 | -.02 | .56 |  |  |  | .58 | .14 | .16 | **<.001** |  |  |
| No of illnesses | -.13 | .09 | -.06 | .13 |  |  |  | .30 | .10 | .11 | **.002** |  |  |
| Model 4 |  |  |  |  | .33 | .33 |  |  |  |  |  | .34 | .22 |
| Age 11 IQ | -.01 | .01 | -.07 | **.04** |  |  |  | -.03 | .01 | -.17 | **<.001** |  |  |
| Age | <.001 | .000 | -.01 | .84 |  |  |  | .001 | .000 | .06 | .06 |  |  |
| Sex | -.01 | .16 | -.003 | .94 |  |  |  | -.29 | .20 | -.05 | .15 |  |  |
| Father’s social class | -.01 | .08 | -.004 | .90 |  |  |  | .01 | .10 | .003 | .94 |  |  |
| Participant’s social class | .07 | .10 | .02 | .49 |  |  |  | .47 | .12 | .13 | **<.001** |  |  |
| No of illnesses | .01 | .07 | .002 | .94 |  |  |  | .16 | .09 | .06 | .07 |  |  |
| Emotional Stability | .10 | .01 | .32 | **<.001** |  |  |  | -.11 | .02 | -.28 | **<.001** |  |  |
| Extraversion | .05 | .01 | .16 | **<.001** |  |  |  | -.03 | .01 | -.07 | **.05** |  |  |
| Agreeableness | .03 | .02 | .07 | .09 |  |  |  | -.07 | .02 | -.12 | **.001** |  |  |
| Conscientiousness | .03 | .01 | .09 | **.01** |  |  |  | -.02 | .02 | -.04 | .21 |  |  |
| Intellect | .02 | .02 | .04 | .25 |  |  |  | -.001 | .02 | -.002 | .95 |  |  |
| Anxiety | -.06 | .03 | -.07 | .08 |  |  |  | .08 | .04 | .08 | **.05** |  |  |
| Depression | -.18 | .04 | -.16 | **<.001** |  |  |  | .16 | .05 | .12 | **.001** |  |  |
| Model 5 |  |  |  |  | .37 | .04 |  |  |  |  |  | .38 | .04 |
| Age 11 IQ | -.02 | .01 | -.12 | **.001** |  |  |  | -.04 | .01 | -.19 | **<.001** |  |  |
| Age | <.001 | .000 | .01 | .79 |  |  |  | .001 | .000 | .06 | .06 |  |  |
| Sex | -.07 | .16 | -.02 | .66 |  |  |  | -.29 | .20 | -.05 | .14 |  |  |
| Father’s social class | -.01 | .08 | -.003 | .92 |  |  |  | .01 | .10 | .002 | .96 |  |  |
| Participant’s social class | .16 | .10 | .06 | .10 |  |  |  | .49 | .12 | .14 | **<.001** |  |  |
| No of illnesses | .04 | .07 | .02 | .60 |  |  |  | .16 | .09 | .06 | .06 |  |  |
| Emotional Stability | .08 | .01 | .25 | **<.001** |  |  |  | -.08 | .02 | -.20 | **<.001** |  |  |
| Extraversion | .05 | .01 | .14 | **<.001** |  |  |  | -.01 | .01 | -.03 | .37 |  |  |
| Agreeableness | .02 | .02 | .03 | .36 |  |  |  | -.06 | .02 | -.11 | **.003** |  |  |
| Conscientiousness | .03 | .01 | .08 | **.02** |  |  |  | -.01 | .02 | -.02 | .54 |  |  |
| Intellect | .02 | .01 | .04 | .24 |  |  |  | .004 | .02 | .01 | .82 |  |  |
| Anxiety | -.04 | .03 | -.05 | .19 |  |  |  | .06 | .04 | .06 | .12 |  |  |
| Depression | -.14 | .04 | -.13 | **<.001** |  |  |  | .11 | .05 | .08 | **.02** |  |  |
| Optimism / Pessimism ^a^ | -.20 | .03 | -.25 | **<.001** |  |  |  | -.31 | .05 | -.24 | **<.001** |  |  |

*Note. n* = 723. Negative adjusted R^2^ values are statistical artefacts which can be considered as zero

*p*-values for significant effects are shown in boldface

^a^ Where optimism is the outcome variable, models were adjusted for pessimism. Where pessimism is the outcome variable, models were adjusted for optimism.

Supplementary Table S2. Multiple linear regression results for older-age IQ predicting optimism and pessimism in the LBC1936.

|  | Optimism | | | | | |  | Pessimism | | | | | |
| --- | --- | --- | --- | --- | --- | --- | --- | --- | --- | --- | --- | --- | --- |
| Model | *B* | *SE B* | *β* | *p* | *Adjusted*  *R^2^* | *∆R^2^* |  | *B* | *SE B* | *β* | *p* | *Adjusted*  *R^2^* | *∆R^2^* |
| Model 1 |  |  |  |  | .000 | .003 |  |  |  |  |  | .11 | .02 |
| Older-age IQ | -.004 | .01 | -.02 | .55 |  |  |  | -.06 | .01 | -.30 | **<.001** |  |  |
| Age | .000 | .000 | -.05 | .14 |  |  |  | .001 | .000 | .11 | **.001** |  |  |
| Sex | -.04 | .17 | -.01 | .82 |  |  |  | -.57 | .20 | -.10 | **.01** |  |  |
| Model 2 |  |  |  |  | -.003 | .001 |  |  |  |  |  | .12 | .02 |
| Older-age IQ | -.01 | .01 | -.03 | .47 |  |  |  | -.05 | .01 | -.25 | **<.001** |  |  |
| Age | .000 | .000 | -.05 | .16 |  |  |  | .001 | .000 | .10 | **.003** |  |  |
| Sex | -.05 | .17 | -.01 | .75 |  |  |  | -.48 | .20 | -.08 | **.02** |  |  |
| Father’s social class | .03 | .10 | .01 | .79 |  |  |  | .02 | .11 | .01 | .87 |  |  |
| Participant’s social class | -.07 | .11 | -.03 | .52 |  |  |  | .55 | .13 | .15 | **<.001** |  |  |
| Model 3 |  |  |  |  | .002 | .002 |  |  |  |  |  | .13 | .01 |
| Older-age IQ | -.01 | .01 | -.03 | .42 |  |  |  | -.05 | .01 | -.24 | **<.001** |  |  |
| Age | .000 | .000 | -.05 | .20 |  |  |  | .001 | .000 | .09 | **.01** |  |  |
| Sex | -.06 | .17 | -.01 | .71 |  |  |  | -.45 | .20 | -.08 | **.03** |  |  |
| Father’s social class | .03 | .10 | .01 | .76 |  |  |  | .01 | .11 | .003 | .92 |  |  |
| Participant’s social class | -.08 | .12 | -.03 | .50 |  |  |  | .56 | .13 | .16 | **<.001** |  |  |
| No of illnesses | -.10 | .08 | -.04 | .24 |  |  |  | .24 | .10 | .09 | **.01** |  |  |
| Model 4 |  |  |  |  | .33 | .33 |  |  |  |  |  | .34 | .21 |
| Older-age IQ | -.02 | .01 | -.12 | **<.001** |  |  |  | -.04 | .01 | -.18 | **<.001** |  |  |
| Age | .000 | .000 | -.01 | .64 |  |  |  | .001 | .000 | .07 | **.01** |  |  |
| Sex | -.05 | .16 | -.01 | .73 |  |  |  | -.36 | .19 | -.06 | .07 |  |  |
| Father’s social class | -.003 | .08 | -.001 | .97 |  |  |  | .05 | .10 | .02 | .62 |  |  |
| Participant’s social class | .06 | .10 | -.02 | .51 |  |  |  | .45 | .12 | .13 | **<.001** |  |  |
| No of illnesses | .02 | .07 | .01 | .78 |  |  |  | .12 | .09 | .04 | .15 |  |  |
| Emotional Stability | .10 | .01 | .32 | **<.001** |  |  |  | -.11 | .02 | -.29 | **<.001** |  |  |
| Extraversion | .05 | .01 | .16 | **<.001** |  |  |  | -.03 | .01 | -.08 | **.02** |  |  |
| Agreeableness | .03 | .02 | .07 | .08 |  |  |  | -.06 | .02 | -.12 | **.002** |  |  |
| Conscientiousness | .03 | .01 | .08 | **.02** |  |  |  | -.01 | .02 | -.03 | .37 |  |  |
| Intellect | .02 | .01 | .05 | .14 |  |  |  | -.003 | .02 | -.01 | .87 |  |  |
| Anxiety | -.06 | .03 | -.08 | .06 |  |  |  | .05 | .04 | .05 | .22 |  |  |
| Depression | -.19 | .04 | -.16 | **<.001** |  |  |  | .16 | .05 | .12 | **.001** |  |  |
| Model 5 |  |  |  |  | .37 | .04 |  |  |  |  |  | .38 | .04 |
| Older-age IQ | -.03 | .01 | -.16 | **<.001** |  |  |  | -.04 | .01 | -.21 | **<.001** |  |  |
| Age | . <.001 | .000 | .01 | .86 |  |  |  | .001 | .000 | .07 | **.02** |  |  |
| Sex | -.13 | .15 | -.03 | .40 |  |  |  | -.37 | .19 | -.06 | **.05** |  |  |
| Father’s social class | .01 | .08 | .003 | .93 |  |  |  | .05 | .09 | .02 | .61 |  |  |
| Participant’s social class | .16 | .09 | .05 | .09 |  |  |  | .47 | .12 | .13 | **<.001** |  |  |
| No of illnesses | .04 | .07 | .02 | .50 |  |  |  | .13 | .08 | .05 | .12 |  |  |
| Emotional Stability | .07 | .01 | .24 | **<.001** |  |  |  | -.08 | .02 | -.21 | **<.001** |  |  |
| Extraversion | .05 | .01 | .14 | **<.001** |  |  |  | -.02 | .01 | -.04 | .26 |  |  |
| Agreeableness | .02 | .02 | .04 | .34 |  |  |  | -.05 | .02 | -.10 | **.01** |  |  |
| Conscientiousness | .03 | .01 | .07 | **.03** |  |  |  | -.01 | .02 | -.01 | .75 |  |  |
| Intellect | .02 | .01 | .05 | .14 |  |  |  | .004 | .02 | .01 | .83 |  |  |
| Anxiety | -.05 | .03 | -.06 | .11 |  |  |  | .03 | .04 | .003 | .44 |  |  |
| Depression | -.15 | .04 | -.13 | **<.001** |  |  |  | .11 | .05 | .07 | **.03** |  |  |
| Optimism / Pessimism ^a^ | -.21 | .03 | -.26 | **<.001** |  |  |  | -.32 | .04 | -.26 | **<.001** |  |  |

*Note.* *n* = 756. Negative adjusted R^2^ values are statistical artefacts which can be considered as zero

*p*-values for significant effects are shown in boldface

^a^ Where optimism is the outcome variable, models were adjusted for pessimism. Where pessimism is the outcome variable, models were adjusted for optimism.

Supplementary Table S3. Multiple linear regression results for IQ change predicting optimism and pessimism in the LBC1936.

|  | Optimism | | | | | |  | Pessimism | | | | | |
| --- | --- | --- | --- | --- | --- | --- | --- | --- | --- | --- | --- | --- | --- |
| Model | *B* | *SE B* | *β* | *p* | *Adjusted*  *R^2^* | *∆R^2^* |  | *B* | *SE B* | *β* | *p* | *Adjusted*  *R^2^* | *∆R^2^* |
| Model 1 |  |  |  |  | -.002 | .002 |  |  |  |  |  | .04 | .03 |
| IQ change | -.07 | .10 | -.03 | .47 |  |  |  | -.44 | .12 | -.13 | **<.001** |  |  |
| Age | .000 | .000 | -.04 | .29 |  |  |  | .001 | .000 | .13 | **.001** |  |  |
| Sex | -.06 | .18 | -.01 | .73 |  |  |  | -.66 | .22 | -.11 | **.003** |  |  |
| Model 2 |  |  |  |  | -.004 | .001 |  |  |  |  |  | .08 | .05 |
| IQ change | -.08 | .10 | -.03 | .44 |  |  |  | -.37 | .12 | -.12 | **.002** |  |  |
| Age | .000 | .000 | -.04 | .32 |  |  |  | .001 | .000 | .11 | **.003** |  |  |
| Sex | -.08 | .18 | -.02 | .67 |  |  |  | -.51 | .22 | -.09 | **.02** |  |  |
| Father’s social class | .01 | .10 | .004 | .93 |  |  |  | .07 | .12 | .02 | .54 |  |  |
| Participant’s social class | -.07 | .11 | -.02 | .53 |  |  |  | .80 | .14 | .22 | **<.001** |  |  |
| Model 3 |  |  |  |  | -.003 | .003 |  |  |  |  |  | .10 | .01 |
| IQ change | -.08 | .10 | -.03 | .44 |  |  |  | -.37 | .12 | -.12 | **.002** |  |  |
| Age | .000 | .000 | -.03 | .40 |  |  |  | .001 | .000 | .10 | **.01** |  |  |
| Sex | -.09 | .18 | -.02 | .62 |  |  |  | -.47 | .21 | -.08 | **.03** |  |  |
| Father’s social class | .01 | .10 | .01 | .90 |  |  |  | .06 | .12 | .02 | .60 |  |  |
| Participant’s social class | -.07 | .11 | -.03 | .53 |  |  |  | .80 | .14 | .22 | **<.001** |  |  |
| No of illnesses | -.12 | .09 | -.05 | .18 |  |  |  | .33 | .10 | .12 | **.001** |  |  |
| Model 4 |  |  |  |  | .33 | .33 |  |  |  |  |  | .32 | .23 |
| IQ change | -.21 | .08 | -.08 | **.01** |  |  |  | -.21 | .10 | -.06 | **.04** |  |  |
| Age | <.001 | .000 | .003 | .92 |  |  |  | .001 | .000 | .07 | **.03** |  |  |
| Sex | -.09 | .16 | -.02 | .57 |  |  |  | -.44 | .21 | -.07 | **.03** |  |  |
| Father’s social class | .01 | .08 | .003 | .92 |  |  |  | .07 | .10 | .02 | .50 |  |  |
| Participant’s social class | .11 | .10 | .04 | .25 |  |  |  | .61 | .12 | .17 | **<.001** |  |  |
| No of illnesses | .02 | .07 | .01 | .76 |  |  |  | .18 | .09 | .06 | **.04** |  |  |
| Emotional Stability | .10 | .01 | .32 | **<.001** |  |  |  | -.11 | .02 | -.28 | **<.001** |  |  |
| Extraversion | .05 | .01 | .16 | **<.001** |  |  |  | -.03 | .02 | -.07 | .07 |  |  |
| Agreeableness | .03 | .02 | .07 | **.05** |  |  |  | -.06 | .02 | -.11 | **.004** |  |  |
| Conscientiousness | .04 | .01 | .09 | **.01** |  |  |  | -.01 | .02 | -.02 | .54 |  |  |
| Intellect | .01 | .01 | .03 | .41 |  |  |  | -.02 | .02 | -.04 | .25 |  |  |
| Anxiety | -.06 | .03 | -.07 | .08 |  |  |  | .09 | .04 | .09 | **.03** |  |  |
| Depression | -.17 | .04 | -.15 | **<.001** |  |  |  | .16 | .05 | .12 | **.001** |  |  |
| Model 5 |  |  |  |  | .37 | .04 |  |  |  |  |  | .36 | .04 |
| IQ change | -.25 | .08 | -.10 | **.002** |  |  |  | -.27 | .10 | -.08 | **.01** |  |  |
| Age | .000 | .000 | .02 | .52 |  |  |  | .001 | .000 | .07 | **.02** |  |  |
| Sex | -.18 | .16 | -.04 | .27 |  |  |  | -.47 | .20 | -.08 | **.02** |  |  |
| Father’s social class | .02 | .08 | .01 | .79 |  |  |  | .07 | .10 | .02 | .47 |  |  |
| Participant’s social class | .23 | .10 | .08 | **.02** |  |  |  | .65 | .12 | .18 | **<.001** |  |  |
| No of illnesses | .06 | .07 | .03 | .42 |  |  |  | .19 | .09 | .07 | **.03** |  |  |
| Emotional Stability | .08 | .01 | .25 | **<.001** |  |  |  | -.08 | .02 | -.21 | **<.001** |  |  |
| Extraversion | .05 | .01 | .14 | **<.001** |  |  |  | -.01 | .01 | -.03 | .42 |  |  |
| Agreeableness | .02 | .02 | .05 | .20 |  |  |  | -.05 | .02 | -.09 | **.01** |  |  |
| Conscientiousness | .03 | .01 | .09 | **.01** |  |  |  | .001 | .02 | .001 | .97 |  |  |
| Intellect | .01 | .01 | .02 | .57 |  |  |  | -.02 | .02 | -.04 | .33 |  |  |
| Anxiety | -.04 | .03 | -.05 | .21 |  |  |  | .07 | .04 | .07 | .07 |  |  |
| Depression | -.14 | .04 | -.13 | **<.001** |  |  |  | .11 | .05 | .08 | **.03** |  |  |
| Optimism / Pessimism ^a^ | -.19 | .03 | -.24 | **<.001** |  |  |  | -.30 | .05 | -.24 | **<.001** |  |  |

*Note.* *n* = 718. Negative adjusted R^2^ values are statistical artefacts which can be considered as zero

*p*-values for significant effects are shown in boldface

^a^ Where optimism is the outcome variable, models were adjusted for pessimism. Where pessimism is the outcome variable, models were adjusted for optimism.

Supplementary Table S4. Multiple linear regression results for older-age IQ adjusted for age-11 IQ predicting optimism and pessimism in the LBC1936.

|  | Optimism | | | | | |  | Pessimism | | | | | |
| --- | --- | --- | --- | --- | --- | --- | --- | --- | --- | --- | --- | --- | --- |
| Model | *B* | *SE B* | *β* | *p* | *Adjusted*  *R^2^* | *∆R^2^* |  | *B* | *SE B* | *β* | *p* | *Adjusted*  *R^2^* | *∆R^2^* |
| Model 1 |  |  |  |  | -.003 | .001 |  |  |  |  |  | .12 | .01 |
| Older-age IQ | -.01 | .01 | -.04 | .48 |  |  |  | -.05 | .01 | -.21 | **<.001** |  |  |
| Age | .000 | .000 | -.04 | .30 |  |  |  | .001 | .000 | .11 | **.003** |  |  |
| Sex | -.07 | .18 | -.01 | .72 |  |  |  | -.50 | .21 | -.08 | **.02** |  |  |
| Age-11 IQ | .01 | .01 | .04 | .51 |  |  |  | -.03 | .01 | -.14 | **.004** |  |  |
| Model 2 |  |  |  |  | -.005 | .000 |  |  |  |  |  | .13 | .02 |
| Older-age IQ | -.01 | .01 | -.04 | .44 |  |  |  | -.04 | .01 | -.19 | **<.001** |  |  |
| Age | .000 | .000 | -.04 | .32 |  |  |  | .001 | .000 | .10 | **.01** |  |  |
| Sex | -.08 | .18 | -.02 | .67 |  |  |  | -.43 | .21 | -.07 | **.04** |  |  |
| Age-11 IQ | .01 | .01 | .03 | .59 |  |  |  | -.02 | .01 | -.11 | **.03** |  |  |
| Father’s social class | .01 | .10 | .003 | .93 |  |  |  | -.02 | .11 | -.01 | .88 |  |  |
| Participant’s social class | -.07 | .12 | -.03 | .55 |  |  |  | .50 | .14 | .14 | **<.001** |  |  |
| Model 3 |  |  |  |  | -.004 | .003 |  |  |  |  |  | .14 | .01 |
| Older-age IQ | -.01 | .01 | -.04 | .43 |  |  |  | -.04 | .01 | -.18 | **<.001** |  |  |
| Age | .000 | .000 | -.03 | .40 |  |  |  | .001 | .000 | .09 | **.01** |  |  |
| Sex | -.09 | .18 | -.02 | .63 |  |  |  | -.40 | .21 | -.07 | .06 |  |  |
| Age-11 IQ | .004 | .01 | .02 | .65 |  |  |  | -.02 | .01 | -.10 | **.05** |  |  |
| Father’s social class | .01 | .10 | .004 | .91 |  |  |  | -.02 | .11 | -.01 | .85 |  |  |
| Participant’s social class | -.08 | .12 | -.03 | .52 |  |  |  | .51 | .14 | .14 | **<.001** |  |  |
| No of illnesses | -.12 | .09 | -.05 | .17 |  |  |  | .27 | .10 | .10 | **.01** |  |  |
| Model 4 |  |  |  |  | .33 | .34 |  |  |  |  |  | .34 | .21 |
| Older-age IQ | -.02 | .01 | -.13 | **.004** |  |  |  | -.03 | .01 | -.12 | **.01** |  |  |
| Age | <.001 | .000 | .002 | .95 |  |  |  | .001 | .000 | .07 | **.03** |  |  |
| Sex | -.06 | .16 | -.01 | .73 |  |  |  | -.34 | .20 | -.06 | .10 |  |  |
| Age-11 IQ | .001 | .01 | .01 | .87 |  |  |  | -.02 | .01 | -.10 | **.03** |  |  |
| Father’s social class | -.02 | .08 | -.01 | .85 |  |  |  | .01 | .10 | .002 | .94 |  |  |
| Participant’s social class | .04 | .10 | .01 | .68 |  |  |  | .43 | .12 | .12 | **.001** |  |  |
| No of illnesses | .01 | .07 | .003 | .92 |  |  |  | .14 | .09 | .05 | .10 |  |  |
| Emotional Stability | .10 | .01 | .32 | **<.001** |  |  |  | -.11 | .02 | -.29 | **<.001** |  |  |
| Extraversion | .05 | .01 | .16 | **<.001** |  |  |  | -.03 | .01 | -.08 | **.03** |  |  |
| Agreeableness | .03 | .02 | .07 | .06 |  |  |  | -.06 | .02 | -.12 | **.002** |  |  |
| Conscientiousness | .03 | .01 | .08 | **.01** |  |  |  | -.02 | .02 | -.04 | .25 |  |  |
| Intellect | .02 | .02 | .05 | .16 |  |  |  | .002 | .02 | .01 | .90 |  |  |
| Anxiety | -.07 | .03 | -.09 | **.04** |  |  |  | .06 | .04 | .06 | .12 |  |  |
| Depression | -.18 | .04 | -.16 | **<.001** |  |  |  | .15 | .05 | .11 | **.002** |  |  |
| Model 5 |  |  |  |  | .38 | .05 |  |  |  |  |  | .39 | .04 |
| Older-age IQ | -.03 | .01 | -.16 | **<.001** |  |  |  | -.03 | .01 | -.15 | **<.001** |  |  |
| Age | .000 | .000 | .02 | .51 |  |  |  | .001 | .000 | .07 | **.02** |  |  |
| Sex | -.13 | .16 | -.03 | .42 |  |  |  | -.35 | .20 | -.06 | .07 |  |  |
| Age-11 IQ | -.003 | .01 | -.02 | .68 |  |  |  | -.02 | .01 | -.09 | **.03** |  |  |
| Father’s social class | -.01 | .08 | -.01 | .86 |  |  |  | .003 | .10 | .001 | .98 |  |  |
| Participant’s social class | .13 | .10 | .05 | .18 |  |  |  | .44 | .12 | .12 | **<.001** |  |  |
| No of illnesses | .04 | .07 | .02 | .58 |  |  |  | .15 | .09 | .05 | .09 |  |  |
| Emotional Stability | .07 | .01 | .24 | **<.001** |  |  |  | -.08 | .02 | -.20 | **<.001** |  |  |
| Extraversion | .04 | .01 | .14 | **<.001** |  |  |  | -.01 | .01 | -.04 | .31 |  |  |
| Agreeableness | .02 | .02 | .04 | .26 |  |  |  | -.05 | .02 | -.10 | **.01** |  |  |
| Conscientiousness | .03 | .01 | .07 | **.02** |  |  |  | -.01 | .02 | -.02 | .60 |  |  |
| Intellect | .02 | .01 | .05 | .14 |  |  |  | .01 | .02 | .02 | .62 |  |  |
| Anxiety | -.05 | .03 | -.07 | .09 |  |  |  | .04 | .04 | .04 | .30 |  |  |
| Depression | -.15 | .04 | -.13 | **<.001** |  |  |  | .09 | .05 | .07 | **.05** |  |  |
| Optimism / Pessimism ^a^ | -.21 | .03 | -.26 | **<.001** |  |  |  | -.32 | .05 | -.26 | **<.001** |  |  |

*Note.* *n* = 718. Negative adjusted R^2^ values are statistical artefacts which can be considered as zero

*p*-values for significant effects are shown in boldface

^a^ Where optimism is the outcome variable, models were adjusted for pessimism. Where pessimism is the outcome variable, models were adjusted for optimism.

Supplementary Table S5. Multiple linear regression results for age-11 IQ predicting optimism and pessimism in the LBC1921.

|  | Optimism | | | | | |  | Pessimism | | | | | |
| --- | --- | --- | --- | --- | --- | --- | --- | --- | --- | --- | --- | --- | --- |
| Model | *B* | *SE B* | *β* | *p* | *Adjusted*  *R^2^* | *∆R^2^* |  | *B* | *SE B* | *β* | *p* | *Adjusted*  *R^2^* | *∆R^2^* |
| Model 1 |  |  |  |  | -.01 | .01 |  |  |  |  |  | .15 | .02 |
| Age-11 IQ | .01 | .01 | .05 | .52 |  |  |  | -.08 | .02 | -.37 | **<.001** |  |  |
| Age | .000 | .001 | -.01 | .87 |  |  |  | .002 | .001 | .11 | .13 |  |  |
| Sex | .45 | .39 | .09 | .26 |  |  |  | -.58 | .43 | -.10 | .18 |  |  |
| Model 2 |  |  |  |  | -.01 | .01 |  |  |  |  |  | .15 | .01 |
| Age-11 IQ | .02 | .02 | .10 | .28 |  |  |  | -.08 | .02 | -.37 | **<.001** |  |  |
| Age | .000 | .001 | -.02 | .77 |  |  |  | .002 | .001 | .11 | .17 |  |  |
| Sex | .40 | .40 | .08 | .32 |  |  |  | -.60 | .44 | -.10 | .17 |  |  |
| Father’s social class | .05 | .21 | .02 | .80 |  |  |  | .24 | .23 | .08 | .29 |  |  |
| Participant’s social class | .27 | .25 | .10 | .28 |  |  |  | -.27 | .27 | -.08 | .32 |  |  |
| Model 3 |  |  |  |  | -.02 | .001 |  |  |  |  |  | .14 | .001 |
| Age-11 IQ | .02 | .02 | .10 | .28 |  |  |  | -.08 | .02 | -.37 | **<.001** |  |  |
| Age | .000 | .001 | -.02 | .79 |  |  |  | .002 | .001 | .11 | .16 |  |  |
| Sex | .41 | .40 | .08 | .31 |  |  |  | -.59 | .44 | -.10 | .18 |  |  |
| Father’s social class | .06 | .21 | .02 | .78 |  |  |  | .25 | .23 | .09 | .28 |  |  |
| Participant’s social class | .26 | .25 | .10 | .30 |  |  |  | -.28 | .27 | -.09 | .31 |  |  |
| No of illnesses | -.08 | .20 | -.03 | .68 |  |  |  | -.11 | .22 | -.04 | .62 |  |  |
| Model 4 |  |  |  |  | .37 | .40 |  |  |  |  |  | .27 | .16 |
| Age-11 IQ | -.01 | .01 | -.07 | .42 |  |  |  | -.06 | .02 | -.29 | **.001** |  |  |
| Age | -.001 | .001 | -.05 | .48 |  |  |  | .002 | .001 | .14 | .06 |  |  |
| Sex | .27 | .34 | .06 | .44 |  |  |  | -.21 | .44 | -.04 | .64 |  |  |
| Father’s social class | .12 | .17 | .05 | .48 |  |  |  | .16 | .21 | .06 | .46 |  |  |
| Participant’s social class | .12 | .20 | .04 | .54 |  |  |  | -.15 | .26 | -.05 | .55 |  |  |
| No of illnesses | -.10 | .16 | -.04 | .53 |  |  |  | -.11 | .21 | -.04 | .58 |  |  |
| Emotional Stability | .12 | .03 | .36 | **<.001** |  |  |  | -.11 | .04 | -.29 | **.003** |  |  |
| Extraversion | .06 | .03 | .16 | **.03** |  |  |  | -.04 | .03 | -.09 | .26 |  |  |
| Agreeableness | -.003 | .04 | -.01 | .93 |  |  |  | -.09 | .05 | -.16 | **.05** |  |  |
| Conscientiousness | .07 | .03 | .16 | **.02** |  |  |  | .04 | .04 | .07 | .36 |  |  |
| Intellect | .07 | .03 | .17 | **.02** |  |  |  | -.04 | .04 | -.09 | .28 |  |  |
| Anxiety | -.03 | .07 | -.04 | .65 |  |  |  | .04 | .09 | .05 | .63 |  |  |
| Depression | -.20 | .07 | -.20 | **.01** |  |  |  | -.02 | .09 | -.02 | .82 |  |  |
| Model 5 |  |  |  |  | .37 | .01 |  |  |  |  |  | .28 | .01 |
| Age-11 IQ | -.02 | .02 | -.09 | .27 |  |  |  | -.06 | .02 | -.30 | **.001** |  |  |
| Age | -.001 | .001 | -.03 | .61 |  |  |  | .002 | .001 | .13 | .07 |  |  |
| Sex | .25 | .34 | .05 | .46 |  |  |  | -.17 | .44 | -.03 | .69 |  |  |
| Father’s social class | .13 | .17 | .06 | .43 |  |  |  | .17 | .21 | .06 | .42 |  |  |
| Participant’s social class | .11 | .20 | .04 | .58 |  |  |  | -.14 | .26 | -.04 | .59 |  |  |
| No of illnesses | -.11 | .16 | -.04 | .50 |  |  |  | -.13 | .21 | -.04 | .54 |  |  |
| Emotional Stability | .11 | .03 | .34 | **<.001** |  |  |  | -.10 | .04 | -.25 | **.02** |  |  |
| Extraversion | .05 | .03 | .15 | **.04** |  |  |  | -.03 | .03 | -.07 | .37 |  |  |
| Agreeableness | -.01 | .04 | -.02 | .78 |  |  |  | -.10 | .05 | -.16 | **.05** |  |  |
| Conscientiousness | .07 | .03 | .17 | **.02** |  |  |  | .04 | .04 | .09 | .26 |  |  |
| Intellect | .07 | .03 | .16 | **.03** |  |  |  | -.04 | .04 | -.07 | .40 |  |  |
| Anxiety | -.03 | .07 | -.04 | .69 |  |  |  | .04 | .09 | .04 | .66 |  |  |
| Depression | -.20 | .07 | -.20 | **.01** |  |  |  | -.05 | .10 | -.04 | .63 |  |  |
| Optimism / Pessimism ^a^ | -.08 | .07 | -.09 | .24 |  |  |  | -.13 | .11 | -.11 | .24 |  |  |

*Note.* *n* = 157. Negative adjusted R^2^ values are statistical artefacts which can be considered as zero

*p*-values for significant effects are shown in boldface

^a^ Where optimism is the outcome variable, models were adjusted for pessimism. Where pessimism is the outcome variable, models were adjusted for optimism.

Supplementary Table S6. Multiple linear regression results for older-age IQ predicting optimism and pessimism in the LBC1921.

|  | Optimism | | | | | |  | Pessimism | | | | | |
| --- | --- | --- | --- | --- | --- | --- | --- | --- | --- | --- | --- | --- | --- |
| Model | *B* | *SE B* | *β* | *p* | *Adjusted*  *R^2^* | *∆R^2^* |  | *B* | *SE B* | *β* | *p* | *Adjusted*  *R^2^* | *∆R^2^* |
| Model 1 |  |  |  |  | -.004 | .01 |  |  |  |  |  | .14 | .03 |
| Older-age IQ | .01 | .01 | .04 | .64 |  |  |  | -.08 | .02 | -.35 | **<.001** |  |  |
| Age | .000 | .001 | -.03 | .75 |  |  |  | .002 | .001 | .11 | .13 |  |  |
| Sex | .49 | .37 | .10 | .18 |  |  |  | -.82 | .42 | -.14 | **.05** |  |  |
| Model 2 |  |  |  |  | -.01 | .01 |  |  |  |  |  | .13 | .002 |
| Older-age IQ | .01 | .01 | .06 | .44 |  |  |  | -.07 | .02 | -.33 | **<.001** |  |  |
| Age | -.001 | .001 | -.04 | .64 |  |  |  | .002 | .001 | .10 | .17 |  |  |
| Sex | .45 | .37 | .10 | .22 |  |  |  | -.85 | .42 | -.14 | **.05** |  |  |
| Father’s social class | .07 | .19 | .03 | .73 |  |  |  | .07 | .22 | .02 | .77 |  |  |
| Participant’s social class | .21 | .23 | .08 | .35 |  |  |  | .12 | .26 | .04 | .64 |  |  |
| Model 3 |  |  |  |  | -.02 | .000 |  |  |  |  |  | .13 | .001 |
| Older-age IQ | .01 | .02 | .06 | .45 |  |  |  | -.07 | .02 | -.33 | **<.001** |  |  |
| Age | -.001 | .001 | -.04 | .64 |  |  |  | .002 | .001 | .10 | .16 |  |  |
| Sex | .45 | .37 | .10 | .23 |  |  |  | -.84 | .43 | -.14 | **.05** |  |  |
| Father’s social class | .07 | .20 | .03 | .74 |  |  |  | .07 | .22 | .02 | .76 |  |  |
| Participant’s social class | .21 | .23 | .08 | .35 |  |  |  | .12 | .26 | .03 | .66 |  |  |
| No of illnesses | .01 | .19 | .002 | .98 |  |  |  | -.08 | .22 | -.03 | .73 |  |  |
| Model 4 |  |  |  |  | .33 | .36 |  |  |  |  |  | .24 | .14 |
| Older-age IQ | -.02 | .01 | -.10 | .16 |  |  |  | -.06 | .02 | -.27 | **<.001** |  |  |
| Age | .000 | .001 | -.01 | .83 |  |  |  | .002 | .001 | .11 | .13 |  |  |
| Sex | .24 | .33 | .05 | .46 |  |  |  | -.32 | .44 | -.05 | .47 |  |  |
| Father’s social class | .09 | .16 | .04 | 59 |  |  |  | .002 | .21 | .001 | .99 |  |  |
| Participant’s social class | .15 | .19 | .05 | .43 |  |  |  | .20 | .25 | .06 | .42 |  |  |
| No of illnesses | -.11 | .16 | -.04 | .50 |  |  |  | .05 | .21 | .02 | .82 |  |  |
| Emotional Stability | .11 | .03 | .35 | **<.001** |  |  |  | -.11 | .04 | -.28 | **.002** |  |  |
| Extraversion | .04 | .02 | .13 | .08 |  |  |  | -.04 | .03 | -.09 | .26 |  |  |
| Agreeableness | -.01 | .04 | -.01 | .90 |  |  |  | -.12 | .05 | -.19 | .**02** |  |  |
| Conscientiousness | .06 | .03 | .14 | **.04** |  |  |  | .02 | .04 | .03 | .68 |  |  |
| Intellect | .07 | .03 | .16 | **.02** |  |  |  | -.06 | .04 | -.11 | .14 |  |  |
| Anxiety | -.02 | .06 | -.02 | .78 |  |  |  | -.001 | .08 | -.001 | .99 |  |  |
| Depression | -.23 | .07 | -.23 | **.001** |  |  |  | -.08 | .09 | -.07 | .37 |  |  |
| Model 5 |  |  |  |  | .35 | .02 |  |  |  |  |  | .26 | .02 |
| Older-age IQ | -.03 | .01 | -.14 | **.05** |  |  |  | -.06 | .02 | -.29 | **<.001** |  |  |
| Age | <.001 | .001 | .002 | .97 |  |  |  | .002 | .001 | .10 | .13 |  |  |
| Sex | .21 | .33 | .04 | .53 |  |  |  | -.27 | .43 | -.05 | .54 |  |  |
| Father’s social class | .09 | .16 | .04 | .58 |  |  |  | .02 | .21 | .01 | .92 |  |  |
| Participant’s social class | .17 | .18 | .06 | .36 |  |  |  | .23 | .24 | .07 | .35 |  |  |
| No of illnesses | -.10 | .16 | -.04 | .52 |  |  |  | .03 | .21 | .01 | .90 |  |  |
| Emotional Stability | .10 | .03 | .31 | **<.001** |  |  |  | -.09 | .04 | -.22 | **.02** |  |  |
| Extraversion | .04 | .02 | .12 | .11 |  |  |  | -.03 | .03 | -.07 | .40 |  |  |
| Agreeableness | -.02 | .04 | -.04 | .60 |  |  |  | -.12 | .05 | -.20 | **.01** |  |  |
| Conscientiousness | .06 | .03 | .15 | **.03** |  |  |  | .03 | .04 | .05 | .46 |  |  |
| Intellect | .06 | .03 | .15 | **.03** |  |  |  | -.04 | .04 | -.08 | .27 |  |  |
| Anxiety | -.02 | .06 | -.02 | .77 |  |  |  | -.004 | .08 | .01 | .96 |  |  |
| Depression | -.24 | .07 | -.24 | **.001** |  |  |  | -.13 | .09 | -.11 | .17 |  |  |
| Optimism / Pessimism ^a^ | -.12 | .06 | -.15 | **.04** |  |  |  | -.21 | .10 | -.17 | **.04** |  |  |

*Note. n* = 172. Negative adjusted R^2^ values are statistical artefacts which can be considered as zero

*p*-values for significant effects are shown in boldface

^a^ Where optimism is the outcome variable, models were adjusted for pessimism. Where pessimism is the outcome variable, models were adjusted for optimism.

Supplementary Table S7. Multiple linear regression results for IQ change predicting optimism and pessimism in the LBC1921.

|  | Optimism | | | | | |  | Pessimism | | | | | |
| --- | --- | --- | --- | --- | --- | --- | --- | --- | --- | --- | --- | --- | --- |
| Model | *B* | *SE B* | *β* | *p* | *Adjusted*  *R^2^* | *∆R^2^* |  | *B* | *SE B* | *β* | *p* | *Adjusted*  *R^2^* | *∆R^2^* |
| Model 1 |  |  |  |  | -.01 | .01 |  |  |  |  |  | .06 | .04 |
| IQ change | .10 | .22 | .04 | .66 |  |  |  | -.62 | .25 | -.19 | **.02** |  |  |
| Age | .000 | .001 | -.03 | .70 |  |  |  | .002 | .001 | .12 | .14 |  |  |
| Sex | .47 | .40 | .10 | .24 |  |  |  | -.92 | .46 | -.16 | **.05** |  |  |
| Model 2 |  |  |  |  | -.02 | .01 |  |  |  |  |  | .07 | .03 |
| IQ change | .10 | .22 | .04 | .65 |  |  |  | -.56 | .25 | -.17 | **.03** |  |  |
| Age | -.001 | .001 | -.04 | .67 |  |  |  | .002 | .001 | .09 | .25 |  |  |
| Sex | .46 | .40 | .09 | .26 |  |  |  | -1.003 | .46 | -.17 | **.03** |  |  |
| Father’s social class | .01 | .21 | .002 | .98 |  |  |  | .40 | .24 | .14 | .09 |  |  |
| Participant’s social class | .21 | .24 | .07 | .39 |  |  |  | .16 | .27 | .05 | .56 |  |  |
| Model 3 |  |  |  |  | -.02 | .000 |  |  |  |  |  | .07 | .001 |
| IQ change | .10 | .22 | .04 | .64 |  |  |  | -.56 | .25 | -.18 | **.03** |  |  |
| Age | -.001 | .001 | -.04 | .66 |  |  |  | .002 | .001 | .10 | .24 |  |  |
| Sex | .46 | .41 | .09 | .26 |  |  |  | -.99 | .47 | -.17 | **.04** |  |  |
| Father’s social class | .004 | .21 | .002 | .98 |  |  |  | .41 | .24 | .14 | .09 |  |  |
| Participant’s social class | .21 | .24 | .08 | .39 |  |  |  | .15 | .28 | .05 | .59 |  |  |
| No of illnesses | .02 | .21 | .01 | .91 |  |  |  | -.09 | .24 | -.03 | .70 |  |  |
| Model 4 |  |  |  |  | .37 | .40 |  |  |  |  |  | .25 | .21 |
| IQ change | -.12 | .18 | -.05 | .50 |  |  |  | -.46 | .24 | -.15 | **.05** |  |  |
| Age | -.001 | .001 | -.05 | .48 |  |  |  | .002 | .001 | .13 | .09 |  |  |
| Sex | .20 | .34 | .04 | .57 |  |  |  | -.50 | .45 | -.09 | .27 |  |  |
| Father’s social class | .11 | .17 | .05 | .52 |  |  |  | .26 | .22 | .09 | .24 |  |  |
| Participant’s social class | .23 | .19 | .09 | .22 |  |  |  | .16 | .25 | .05 | .52 |  |  |
| No of illnesses | -.05 | .17 | -.02 | .76 |  |  |  | -.04 | .22 | -.01 | .87 |  |  |
| Emotional Stability | .11 | .03 | .34 | **<.001** |  |  |  | -.13 | .04 | -.33 | **.001** |  |  |
| Extraversion | .06 | .03 | .18 | **.02** |  |  |  | -.03 | .03 | -.06 | .44 |  |  |
| Agreeableness | -.01 | .04 | -.02 | .80 |  |  |  | -.11 | .05 | -.18 | **.04** |  |  |
| Conscientiousness | .06 | .03 | .15 | **.05** |  |  |  | .01 | .04 | .03 | .72 |  |  |
| Intellect | .07 | .03 | .16 | **.02** |  |  |  | -.09 | .04 | -.18 | **.02** |  |  |
| Anxiety | -.03 | .07 | -.04 | .66 |  |  |  | .05 | .09 | .05 | .59 |  |  |
| Depression | -.22 | .07 | -.22 | **.004** |  |  |  | -.09 | .10 | -.08 | .34 |  |  |
| Model 5 |  |  |  |  | .37 | .01 |  |  |  |  |  | .25 | .01 |
| IQ change | -.16 | .18 | -.06 | .38 |  |  |  | -.48 | -.24 | -.15 | **.05** |  |  |
| Age | -.001 | .001 | -.04 | .61 |  |  |  | .002 | .001 | .12 | .11 |  |  |
| Sex | .15 | .34 | .03 | .66 |  |  |  | -.47 | .45 | -.08 | .30 |  |  |
| Father’s social class | .13 | .17 | .06 | .44 |  |  |  | .27 | .22 | .10 | .21 |  |  |
| Participant’s social class | .25 | .19 | .09 | .20 |  |  |  | .20 | .25 | .06 | .43 |  |  |
| No of illnesses | -.05 | .17 | -.02 | .75 |  |  |  | -.04 | .22 | -.02 | .84 |  |  |
| Emotional Stability | .10 | .03 | .31 | **.001** |  |  |  | -.12 | .04 | -.29 | **.01** |  |  |
| Extraversion | .06 | .03 | .17 | **.02** |  |  |  | -.02 | .03 | -.04 | .62 |  |  |
| Agreeableness | -.02 | .04 | -.04 | .63 |  |  |  | -.11 | .05 | -.18 | **.03** |  |  |
| Conscientiousness | .06 | .03 | .15 | **.04** |  |  |  | .02 | .04 | .05 | .56 |  |  |
| Intellect | .06 | .03 | .14 | **.05** |  |  |  | -.08 | .04 | -.16 | **.03** |  |  |
| Anxiety | -.03 | .07 | -.03 | .71 |  |  |  | .05 | .09 | .05 | .62 |  |  |
| Depression | -.23 | .07 | -.23 | **.003** |  |  |  | -.13 | .10 | -.11 | .21 |  |  |
| Optimism / Pessimism^a^ | -.09 | .07 | -.10 | .18 |  |  |  | -.15 | .11 | -.12 | .18 |  |  |

*Note.* *n* = 152. Negative adjusted R^2^ values are statistical artefacts which can be considered as zero

*p*-values for significant effects are shown in boldface

^a^ Where optimism is the outcome variable, models were adjusted for pessimism. Where pessimism is the outcome variable, models were adjusted for optimism.

Supplementary Table S8. Multiple linear regression results for older-age IQ adjusted for age-11 IQ predicting optimism and pessimism in the LBC1921.

|  | Optimism | | | | | |  | Pessimism | | | | | |
| --- | --- | --- | --- | --- | --- | --- | --- | --- | --- | --- | --- | --- | --- |
| Model | *B* | *SE B* | *β* | *p* | *Adjusted*  *R^2^* | *∆R^2^* |  | *B* | *SE B* | *β* | *p* | *Adjusted*  *R^2^* | *∆R^2^* |
| Model 1 |  |  |  |  | -.01 | .001 |  |  |  |  |  | .20 | .05 |
| Older-age IQ | .01 | .02 | .05 | .62 |  |  |  | -.06 | .02 | -.26 | **.002** |  |  |
| Age | -.001 | .001 | -.03 | .69 |  |  |  | .002 | .001 | .14 | .07 |  |  |
| Sex | .43 | .41 | .09 | .29 |  |  |  | -.56 | .43 | -.10 | .20 |  |  |
| Age-11 IQ | .01 | .02 | .03 | .77 |  |  |  | -.05 | .02 | -.24 | **.004** |  |  |
| Model 2 |  |  |  |  | -.01 | .01 |  |  |  |  |  | .20 | .01 |
| Older-age IQ | .01 | .02 | .06 | .54 |  |  |  | -.06 | .02 | -.26 | **.003** |  |  |
| Age | -.001 | .001 | -.05 | .58 |  |  |  | .002 | .001 | .13 | .08 |  |  |
| Sex | .36 | .41 | .07 | .38 |  |  |  | -.56 | .44 | -.10 | .21 |  |  |
| Age-11 IQ | .01 | .02 | .07 | .47 |  |  |  | -.06 | .02 | -.26 | **.004** |  |  |
| Father’s social class | .06 | .21 | .03 | .77 |  |  |  | .14 | .23 | .05 | .55 |  |  |
| Participant’s social class | .30 | .25 | .11 | .23 |  |  |  | -.28 | .27 | -.09 | .30 |  |  |
| Model 3 |  |  |  |  | -.02 | .000 |  |  |  |  |  | .19 | .001 |
| Older-age IQ | .01 | .02 | .06 | .54 |  |  |  | -.06 | .02 | -.26 | **.003** |  |  |
| Age | -.001 | .001 | -.05 | .58 |  |  |  | .002 | .001 | .13 | .08 |  |  |
| Sex | .36 | .41 | .07 | .39 |  |  |  | -.55 | .44 | -.09 | .22 |  |  |
| Age-11 IQ | .01 | .02 | .07 | .47 |  |  |  | -.06 | .02 | -.26 | **.004** |  |  |
| Father’s social class | .06 | .22 | .03 | .77 |  |  |  | .14 | .23 | .05 | .54 |  |  |
| Participant’s social class | .30 | .06 | .11 | .24 |  |  |  | -.29 | .22 | -.02 | .75 |  |  |
| No of illnesses | .02 | .21 | .01 | .93 |  |  |  | -.07 | .22 | -.05 | .75 |  |  |
| Model 4 |  |  |  |  | .37 | .40 |  |  |  |  |  | .31 | .15 |
| Older-age IQ | -.01 | .01 | -.07 | .41 |  |  |  | -.05 | .02 | -.21 | **.01** |  |  |
| Age | -.001 | .001 | -.05 | .51 |  |  |  | .003 | .001 | .14 | **.05** |  |  |
| Sex | .26 | .35 | .05 | .46 |  |  |  | -.21 | .44 | -.04 | .64 |  |  |
| Age-11 IQ | -.01 | .02 | -.05 | .58 |  |  |  | -.04 | .02 | -.21 | **.03** |  |  |
| Father’s social class | .07 | .17 | .03 | .69 |  |  |  | .07 | .21 | .03 | .73 |  |  |
| Participant’s social class | .16 | .20 | .06 | .42 |  |  |  | -.16 | .25 | -.05 | .53 |  |  |
| No of illnesses | -.05 | .17 | -.02 | .77 |  |  |  | -.03 | .21 | -.01 | .91 |  |  |
| Emotional Stability | .12 | .03 | .35 | **<.001** |  |  |  | -.12 | .04 | -.29 | **.003** |  |  |
| Extraversion | .06 | .03 | .16 | **.03** |  |  |  | -.05 | .03 | -.11 | .16 |  |  |
| Agreeableness | -.01 | .04 | -.02 | .84 |  |  |  | -.10 | 0.5 | -.16 | **.04** |  |  |
| Conscientiousness | .06 | .03 | .15 | **.05** |  |  |  | .02 | .04 | .04 | .62 |  |  |
| Intellect | .08 | .03 | .19 | **.01** |  |  |  | -.03 | .04 | -.03 | .42 |  |  |
| Anxiety | -.04 | .07 | -.04 | .63 |  |  |  | .04 | .09 | .04 | .70 |  |  |
| Depression | -.22 | .07 | -.22 | .004 |  |  |  | -.08 | .09 | -.07 | .37 |  |  |
| Model 5 |  |  |  |  | .38 | .01 |  |  |  |  |  | .32 | .01 |
| Older-age IQ | -.02 | .01 | -.10 | .24 |  |  |  | -.05 | .02 | -.22 | **.01** |  |  |
| Age | .000 | .001 | -.03 | .71 |  |  |  | .002 | .001 | .13 | .06 |  |  |
| Sex | .23 | .35 | .05 | .50 |  |  |  | -.16 | .43 | -.03 | .72 |  |  |
| Age-11 IQ | -.01 | .02 | -.08 | .38 |  |  |  | -.05 | .02 | -.21 | **.02** |  |  |
| Father’s social class | .08 | .17 | .03 | .65 |  |  |  | .09 | .21 | .03 | .69 |  |  |
| Participant’s social class | .15 | .20 | .05 | .47 |  |  |  | -.13 | .25 | -.04 | .61 |  |  |
| No of illnesses | -.05 | .16 | -.02 | .76 |  |  |  | -.03 | .21 | -.01 | .87 |  |  |
| Emotional Stability | .10 | .03 | .31 | **.001** |  |  |  | -.09 | .04 | -.24 | **.02** |  |  |
| Extraversion | .05 | .03 | .15 | **.05** |  |  |  | -.04 | .03 | -.09 | .28 |  |  |
| Agreeableness | -.02 | .04 | -.04 | .61 |  |  |  | -.10 | .05 | -.17 | **.04** |  |  |
| Conscientiousness | .06 | .03 | .16 | **.04** |  |  |  | .03 | .04 | .06 | .43 |  |  |
| Intellect | .08 | .03 | .18 | **.02** |  |  |  | -.02 | .04 | -.03 | .67 |  |  |
| Anxiety | -.03 | .07 | -.04 | .67 |  |  |  | .03 | .09 | .03 | .75 |  |  |
| Depression | -.23 | .07 | -.23 | **.003** |  |  |  | -.12 | .10 | -.11 | .20 |  |  |
| Optimism / Pessimism^a^ | -.12 | .07 | -.14 | .08 |  |  |  | -.19 | .11 | -.15 | .08 |  |  |

*Note.* *n* = 152. Negative adjusted R^2^ values are statistical artefacts which can be considered as zero

*p*-values for significant effects are shown in boldface

^a^ Where optimism is the outcome variable, models were adjusted for pessimism. Where pessimism is the outcome variable, models were adjusted for optimism.

Supplementary Table S9. Multiple linear regression results for three cognitive ability measures predicting pessimism for males and females in the LBC1936.

|  |  | Males | | | | | |  | Females | | | | | |
| --- | --- | --- | --- | --- | --- | --- | --- | --- | --- | --- | --- | --- | --- | --- |
| Cognitive predictor  (*n male / female*) | Model | *B* | *SE B* | *β* | *p* | *Adjusted R^2^* | *∆R^2^* |  | *B* | *SE B* | *β* | *p* | *Adjusted R^2^* | *∆R^2^* |
| Age-11 IQ (*n* =369 / 354 ) |  |  |  |  |  |  |  |  |  |  |  |  |  |  |
|  | 1 | -.06 | .01 | -.28 | **<.001** | .10 | .02 |  | -.06 | .01 | -.29 | **<.001** | .08 | .001 |
|  | 2 | -.04 | .01 | -.22 | **<.001** | .12 | .02 |  | -.05 | .01 | -.24 | **<.001** | .09 | .02 |
|  | 3 | -.04 | .01 | -.21 | **<.001** | .13 | .01 |  | -.05 | .01 | -.23 | **<.001** | .10 | .01 |
|  | 4 | -.03 | .01 | -.15 | **.001** | .41 | .29 |  | -.04 | .01 | -.19 | **<.001** | .27 | .18 |
| Older-age IQ (*n* = 385 / 371) |  |  |  |  |  |  |  |  |  |  |  |  |  |  |
|  | 1 | -.07 | .01 | -.31 | **<.001** | .12 | .03 |  | -.06 | .01 | -.28 | **<.001** | .08 | .004 |
|  | 2 | -.05 | .01 | -.26 | **<.001** | .14 | .02 |  | -.05 | .01 | -.24 | **<.001** | .09 | .02 |
|  | 3 | -.05 | .01 | -.25 | **<.001** | .14 | .01 |  | -.05 | .01 | -.23 | **<.001** | .10 | .004 |
|  | 4 | -.04 | .01 | -.17 | **<.001** | .40 | .26 |  | -.04 | .01 | -.18 | **<.001** | .26 | .18 |
|  | 5 | -.03 | .01 | -.13 | **.02** | .41 | .27 |  | -.02 | .02 | -.10 | .12 | .27 | .18 |
| IQ change (*n* =366 / 352) |  |  |  |  |  |  |  |  |  |  |  |  |  |  |
|  | 1 | -.48 | .16 | -.15 | **.003** | .05 | .03 |  | -.36 | .18 | -.11 | **.05** | .01 | .01 |
|  | 2 | -.41 | .16 | -.13 | **.01** | .09 | .05 |  | -.31 | .18 | -.09 | .09 | .05 | .05 |
|  | 3 | -.41 | .16 | -.13 | **.01** | .11 | .02 |  | -.31 | .18 | -.09 | .09 | .06 | .01 |
|  | 4 | -.25 | .13 | -.08 | .06 | .39 | .29 |  | -.17 | .16 | -.05 | .30 | .24 | .20 |

*Note.* Model 1 = adjusted for age and sex; Model 2 = as model 1 and additionally adjusted for father’s and participant’s social class; Model 3 = as model 2 and additionally adjusted for total number of illnesses; Model 4 = as model 3 and additionally adjusted for personality and mood. Model 5 = adjusted for age, sex, age-11 IQ, father’s and participant’s social class, total number of illnesses, personality and mood. *p*-values for significant effects are shown in boldface
